# Targeting mechanosensitive MDM4 promotes lung fibrosis resolution in aged mice

**DOI:** 10.1084/jem.20202033

**Published:** 2021-03-10

**Authors:** Jing Qu, Shan-Zhong Yang, Yi Zhu, Ting Guo, Victor J. Thannickal, Yong Zhou

**Affiliations:** 1 Department of Medicine, Division of Pulmonary, Allergy and Critical Care Medicine, University of Alabama at Birmingham, Birmingham, AL; 2 Department of Pathophysiology, School of Basic Medicine, Tongji Medical College, Huazhong University of Science and Technology, Wuhan, Hubei, China; 3 The Second Xiangya Hospital, Central-South University, Changsha, Hunan, China

## Abstract

Aging is a strong risk factor and an independent prognostic factor for progressive human idiopathic pulmonary fibrosis (IPF). Aged mice develop nonresolving pulmonary fibrosis following lung injury. In this study, we found that mouse double minute 4 homolog (MDM4) is highly expressed in the fibrotic lesions of human IPF and experimental pulmonary fibrosis in aged mice. We identified MDM4 as a matrix stiffness–regulated endogenous inhibitor of p53. Reducing matrix stiffness down-regulates MDM4 expression, resulting in p53 activation in primary lung myofibroblasts isolated from IPF patients. Gain of p53 function activates a gene program that sensitizes lung myofibroblasts to apoptosis and promotes the clearance of apoptotic myofibroblasts by macrophages. Destiffening of the fibrotic lung matrix by targeting nonenzymatic cross-linking or genetic ablation of Mdm4 in lung (myo)fibroblasts activates the Mdm4–p53 pathway and promotes lung fibrosis resolution in aged mice. These findings suggest that mechanosensitive MDM4 is a molecular target with promising therapeutic potential against persistent lung fibrosis associated with aging.

## Teaser

Aging is associated with the development of human idiopathic pulmonary fibrosis (IPF), a lethal fibrotic lung disease. In this study, the investigators identify a novel molecular target with therapeutic potential against age-related persistent lung fibrosis in mice.

## Graphical Abstract

**Figure:**
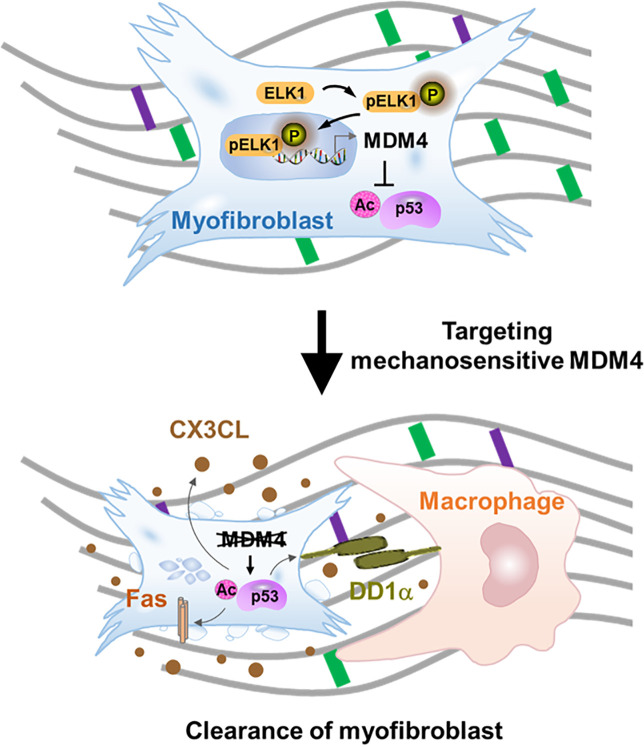

## Introduction

Human idiopathic pulmonary fibrosis (IPF) is a progressive, lethal fibrotic lung disease that primarily affects older adults (Castriotta et al., 2010; Naik and Moore, 2010; Rojas et al., 2015; Selman and Pardo, 2014; Sueblinvong et al., 2012; Thannickal, 2013). Aged mice develop nonresolving pulmonary fibrosis in response to bleomycin-induced lung injury, whereas lung fibrosis in younger mice resolves over time (Hecker et al., 2014). The clinical observations and investigations of lung fibrosis in animal models suggest that aging is associated with the development of persistent/progressive pulmonary fibrosis.

Matrix stiffening is a prominent feature of lung fibrosis (Booth et al., 2012; Liu et al., 2010; Zhou et al., 2020). The formation of intra- and intermolecular cross-links in the extracellular matrix (ECM), in particular collagenous ECM, by enzyme- and non-enzyme-mediated cross-linking reactions is a crucial factor that stiffens the ECM (Barry-Hamilton et al., 2010; Greenwald, 2007; Olsen et al., 2011; Simm, 2013; Verzijl et al., 2002; Wells, 2008). Highly cross-linked collagens are resistant to proteolytic degradation, further stabilizing the fibrotic ECM (DeGroot et al., 2001; Grenard et al., 2001; Locy et al., 2020; Mott et al., 1997). Accumulating evidence indicates that mechanical interactions between (myo)fibroblasts and the stiffened ECM provide a feedforward mechanism that sustains and/or perpetuates pulmonary fibrosis (Chen et al., 2016; Fiore et al., 2015; Liu et al., 2015; Liu et al., 2010; Qu et al., 2018; Rahaman et al., 2014; Southern et al., 2016; Wipff et al., 2007; Zhou et al., 2013; Zhou et al., 2020). Targeting matrix stiffness to disrupt the mechano-fibrogenic feedback loop is a promising strategy for treatment of persistent and progressive lung fibrosis.

The lung is an organ with the capacity of resolving fibrotic repair and reinstatement of tissue homeostasis (Beers and Morrisey, 2011; El Agha et al., 2017; Glasser et al., 2016; Horowitz and Thannickal, 2019; Islam et al., 2012; Jun and Lau, 2018; Kheirollahi et al., 2019; Rangarajan et al., 2018). Lung fibrosis resolution is thought to involve degradation of excessive ECM, removal of myofibroblasts (effectors of tissue fibrosis), and regeneration of normal lung tissue by stem cells (Atabai et al., 2020; Glasser et al., 2016; Horowitz and Thannickal, 2019). Mechanisms underlying the reversal of lung fibrosis remain poorly understood. p53 is a tumor suppressor and sequence-specific transcription factor that directly regulates ∼500 target genes, thereby controlling a broad range of cellular processes in both malignant and nontransformed cells (Haupt et al., 2003). In the normal wound-healing process, p53 expression is initially suppressed and reemerges in the healing phase, reaching the peak level at the completion of reepithelialization (Antoniades et al., 1994). In contrast, myofibroblasts, which are effectors of tissue fibrosis, emerge in response to tissue injury and undergo apoptosis at the wound closure (Desmoulière et al., 1995). These observations suggest an inverse correlation between p53 expression and the presence of myofibroblasts during tissue repair after injury. Previous studies have shown that (myo)fibroblasts in IPF lungs express a reduced level of p53 compared with normal fibroblasts in the control subjects (Akram et al., 2014; Cisneros et al., 2012). It is currently not known whether p53 regulates the fate decision of lung myofibroblasts and whether myofibroblast gaining p53 function impacts repair of the injured lungs.

Physiological activation of p53 occurs by the release of p53 from endogenous inhibitors, a process known as antirepression or derepression (Kruse and Gu, 2009). MDM2 and MDM4 (also known as HDMX and MDMX) are the two major endogenous inhibitors of p53 (Finch et al., 2002; Parant et al., 2001; Shvarts et al., 1996; Wade et al., 2013). MDM2 functions to promote degradation of p53 with its intrinsic E3-ligase activity (Barboza et al., 2008; Toledo et al., 2006). MDM4 does not have E3-ligase activity (Jackson and Berberich, 2000; Stad et al., 2001). Instead, it binds the transactivation domain of p53, thereby compromising the transcriptional function of p53 (Francoz et al., 2006; Sabbatini and McCormick, 2002; Shvarts et al., 1996).

In this study, we observed that MDM4 is highly expressed in the fibrotic lesions of both human IPF and bleomycin-induced experimental lung fibrosis in aged mice. We identified MDM4 as a matrix stiffness–regulated negative regulator of p53. In vitro studies demonstrated that reducing matrix stiffness activates a MDM4–p53-dependent gene program, which sensitizes lung myofibroblasts to apoptosis, recruits macrophages through the release of a paracrine signal, and promotes macrophage-mediated efferocytosis of apoptotic myofibroblasts. Destiffening of the fibrotic ECM by targeting nonenzymatic glycation cross-linking or genetic ablation of *Mdm4* in collagen I–producing (myo)fibroblasts reverses persistent lung fibrosis in aged mice.

## Results

### MDM4 expression is increased in human IPF and bleomycin-induced pulmonary fibrosis in aged mice

**Figure 1.**
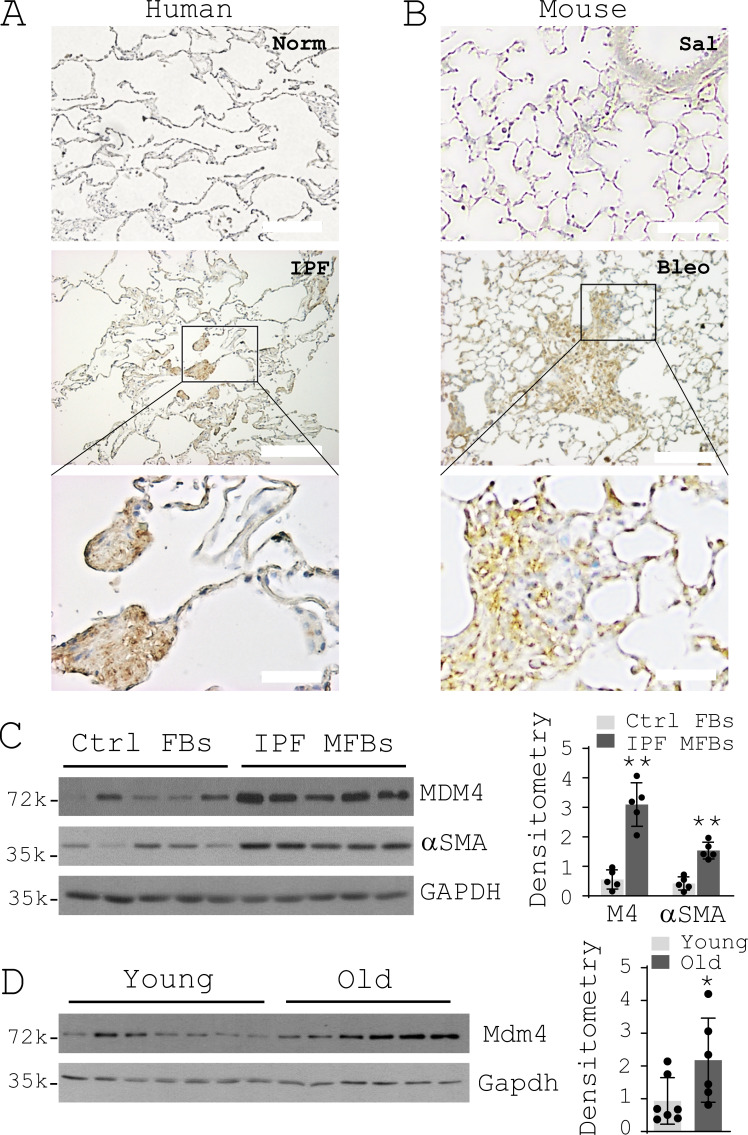
**MDM4 expression is increased in human IPF and bleomycin-induced lung fibrosis in aged mice. (A and B)** IHC analysis of MDM4 expression in formalin-fixed, paraffin-embedded lung tissue sections derived from patients with IPF and normal (Norm) human donors (A; *n* = 3 per group) and from aged mice treated with bleomycin (Bleo) or saline (Sal; B; *n *= 3 per group). Representative human IHC images were derived from a 63-yr-old white male (IPF) and a 52-yr-old white male (Norm). Scale bar = 50 µm (bottom two images) or 200 µm (top four images). **(C) **Immunoblot analysis of MDM4 expression in primary lung (myo)fibroblasts isolated from patients with IPF or normal control (Ctrl) subjects (*n* = 5 per group). **(D)** Immunoblot analysis of Mdm4 expression in primary Pdgfrα+ lung fibroblasts isolated from aged (*n* = 6) and young mice (*n* = 7) by flow cytometry. Relative levels of MDM4 protein were determined by scanning densitometry of the blots and normalized to GAPDH expression. Results are the means ± SD of three separate experiments. *, P < 0.05; **, P < 0.01 (ANOVA). FB, fibroblasts; MFB, myofibroblasts; k, kilodalton.

**Figure S1.**
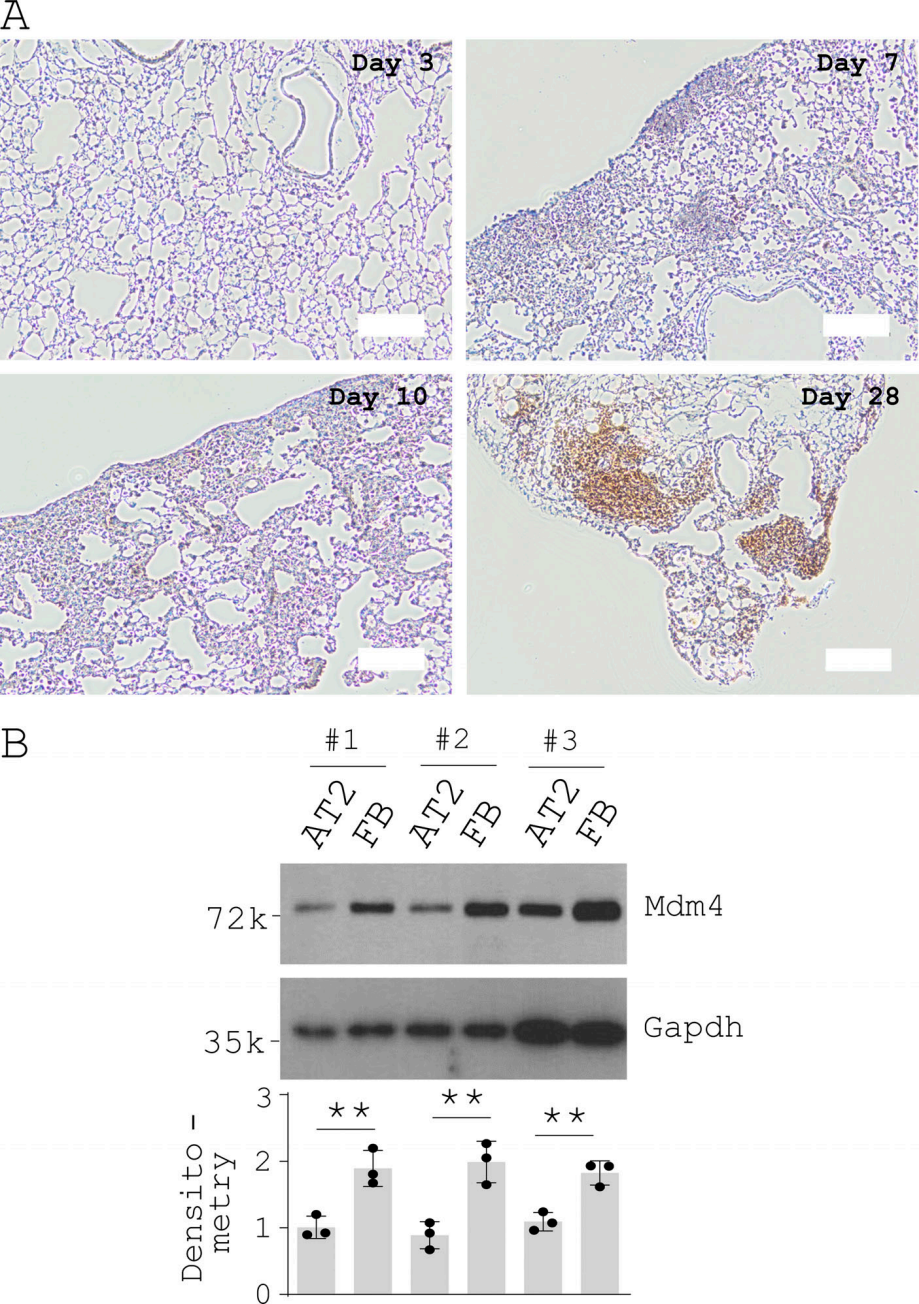
**Mdm4 expression during the course of bleomycin-induced lung fibrosis in aged mice and comparision of Mdm4 expression between uncultured lung fibroblasts and alveolar epithelial cells isolated from aged mice. (A)** IHC analysis of Mdm4 expression during the course of bleomycin-induced lung fibrosis in aged mice (*n* = 3 per time points). Representative IHC images are shown. Scale bars = 100 µm. **(B)** Immunoblot analysis of Mdm4 expression for uncultured primary lung fibroblasts (FB) and AT2 cells (purified by FACS) from aged mice (*n* = 3 mice). Relative levels of Mdm4 protein were determined by scanning densitometry of the blots and normalized to GAPDH expression. Results are the means ± SD of three separate experiments, each performed in triplicate. **, P < 0.01 (ANOVA). k, kilodalton.

To evaluate the levels of MDM4 expression in normal versus fibrotic lungs, we performed immunohistochemical (IHC) analysis on formalin-fixed, paraffin-embedded lung tissues harvested from patients with IPF, failed human donors, and aged (15-mo-old) mice subjected to intratracheal bleomycin or saline treatment. MDM4-positive signals were observed in the fibrotic lesions of both human and mouse lungs, whereas little or no MDM4 expression was found in the relatively normal area of the fibrotic lungs and the normal control lungs (Fig. 1, A and B). Mdm4 expression in bleomycin-treated mouse lungs was time dependent, with minimal expression at day 3 (acute lung injury), increasing expression at days 7 and 10 (lung inflammation), and highest expression at day 28 (lung fibrosis; Fig. S1 A). Primary lung fibroblasts isolated from patients with IPF expressed higher levels of MDM4 and αSMA than cells isolated from the control subjects (Fig. 1 C). Uncultured primary Pdgfrα^+^ lung fibroblasts isolated from aged mice showed higher levels of Mdm4 expression than cells isolated from young (8-wk-old) mice (Fig. 1 D). Additionally, uncultured Pdgfrα^+^ lung fibroblasts expressed relatively higher levels of Mdm4 than uncultured type II alveolar epithelial (AT2) cells isolated from the same mouse (Fig. S1 B). These data suggest that MDM4 expression is increased in lung myofibroblasts in human IPF and experimental lung fibrosis, and the expression is associated with aging.

### MDM4 is a matrix stiffness–regulated negative regulator of p53

**Figure 2.**
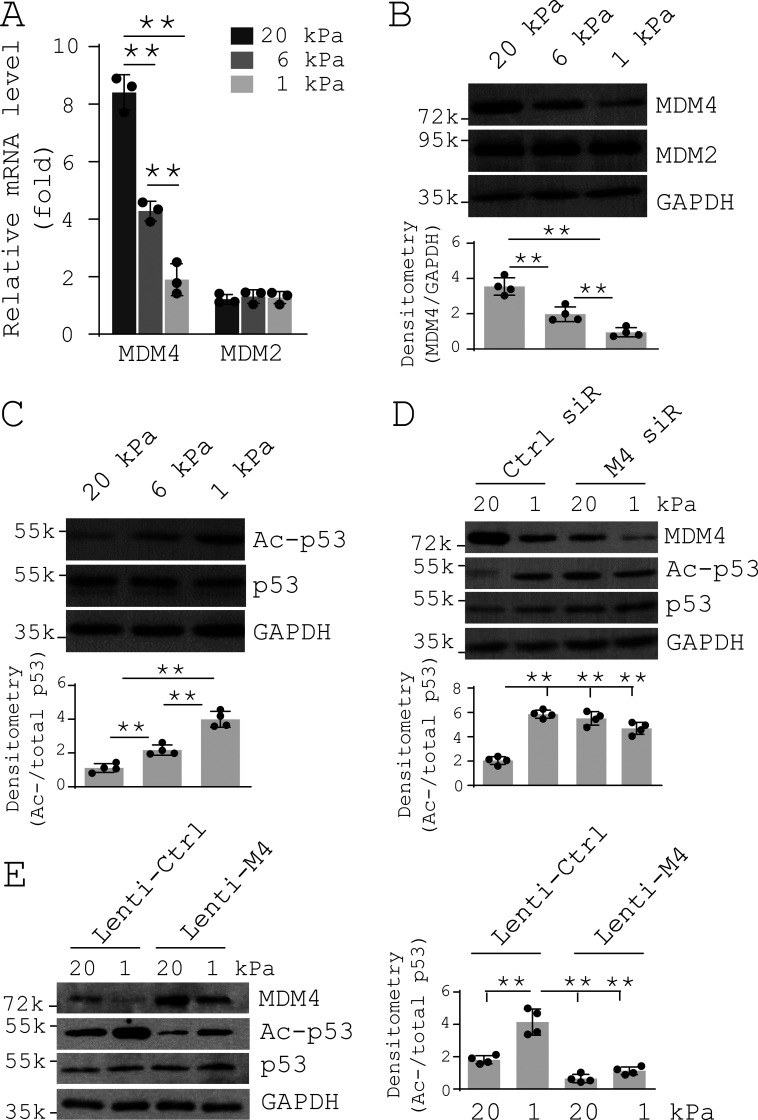
**MDM4 is a matrix stiffness–regulated mechanosensitive inhibitor of p53.** Primary human lung fibroblasts were cultured on 1–20-kPa PA gels. **(A)** mRNA levels of MDM4 and MDM2 were determined by qPCR. **(B)** Protein levels of MDM4 and MDM2 were determined by immunoblot. **(C)** Levels of acetylated p53 (Ac-p53) and total p53 were determined by immunoblot. **(D)** Human lung fibroblasts were cultured on 1- or 20-kPa PA gels in the presence of 50 nM MDM4 (M4) siRNAs (siR) or control siRNAs. Levels of MDM4, Ac-p53, and total p53 were determined by immunoblot. **(E)** Human lung fibroblasts infected with MDM4-expressing lentivirus (Lenti-M4) or the control lentivirus (Lenti-Ctrl) were cultured on 1- or 20-kPa PA gels. Levels of MDM4, Ac-p53, and total p53 were determined by immunoblot. GAPDH was used as internal reference control in qPCR analysis and as loading control in immunoblot analysis. Results are the means ± SD of three or four separate experiments. **, P < 0.01 (ANOVA). k, kilodalton.

**Figure S2.**
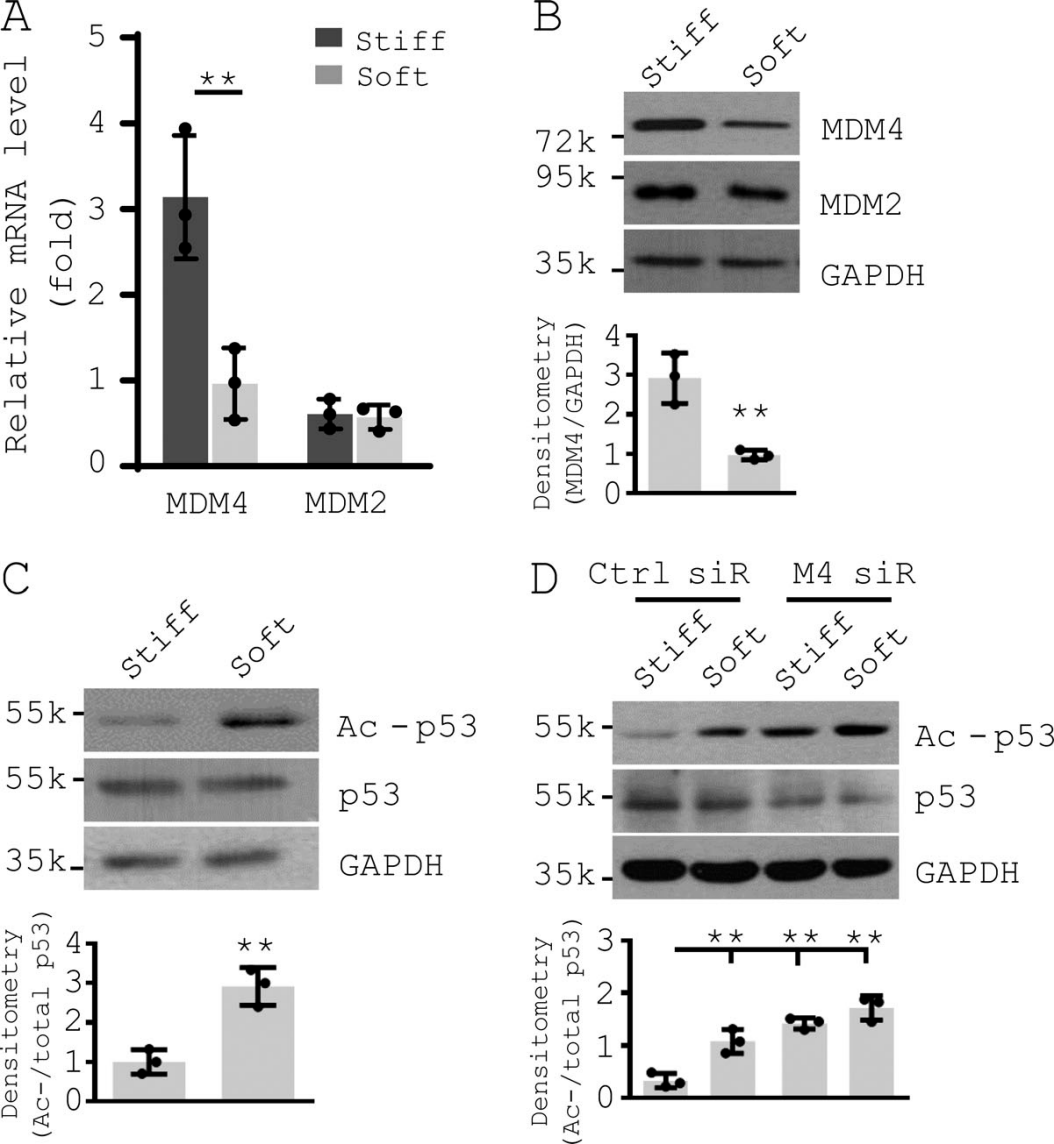
**MDM4 is a matrix stiffness–regulated mechanosensitive inhibitor of p53.** Primary human lung fibroblasts were cultured on decellularized lung fibroblast ECM treated with 2% genipin (stiff) or 0% genipin (soft; Qu et al., 2018; Subbiah et al., 2016). **(A)** mRNA levels of MDM4 and MDM2 were determined by qPCR. **(B)** Protein levels of MDM4 and MDM2 were determined by immunoblot. **(C)** Levels of acetylated p53 (Ac-p53) and total p53 were determined by immunoblot. **(D)** Human lung fibroblasts were cultured on soft or stiff ECM in the presence of 50 nM MDM4 (M4) siRNAs (siR) or the control siRNAs. Levels of MDM4, Ac-p53, and total p53 were determined by immunoblot. GAPDH was used as internal reference control in qPCR analysis and as loading control in immunoblot analysis. Results are the means ± SD of three separate experiments, each performed in triplicate. **, P < 0.01 (ANOVA). k, kilodalton.

Matrix stiffness has been identified as a crucial mechanical factor that regulates lung fibrogenesis (Tschumperlin et al., 2018; Zhou et al., 2018). To determine the potential mechanisms by which MDM4 expression is up-regulated in the fibrotic lungs, we cultured primary human lung fibroblasts on polyacrylamide (PA) matrix substrates with varying stiffness (1–20 kPa) that spanned a physiological stiffness range of normal to fibrotic lungs (Booth et al., 2012; Liu et al., 2010; Zhou et al., 2020). We observed that stiffening of the matrix substrates increased MDM4 expression at both the mRNA and protein level in lung fibroblasts (Fig. 2, A and B). In contrast, changes of matrix stiffness did not alter MDM2 expression. Matrix stiffening decreased the level of acetylated p53 (active p53) without changing expression of total p53 (Fig. 2 C). Knockdown of MDM4 by siRNAs resulted in an increase in p53 acetylation/activation under stiff matrix conditions (Fig. 2 D). Overexpression of MDM4 by lentiviral vectors inhibited p53 acetylation/activation under both soft and stiff matrix conditions (Fig. 2 E). Furthermore, we confirmed matrix stiffness–regulated MDM4 expression and p53 acetylation by a second approach (Fig. S2) utilizing a stiffness-tunable, lung fibroblast–derived biological ECM system (Qu et al., 2018). Together, these data suggest that MDM4 is a matrix stiffness–regulated endogenous inhibitor of p53.

### ELK1 mediates matrix stiffness–regulated MDM4 expression

**Figure 3.**
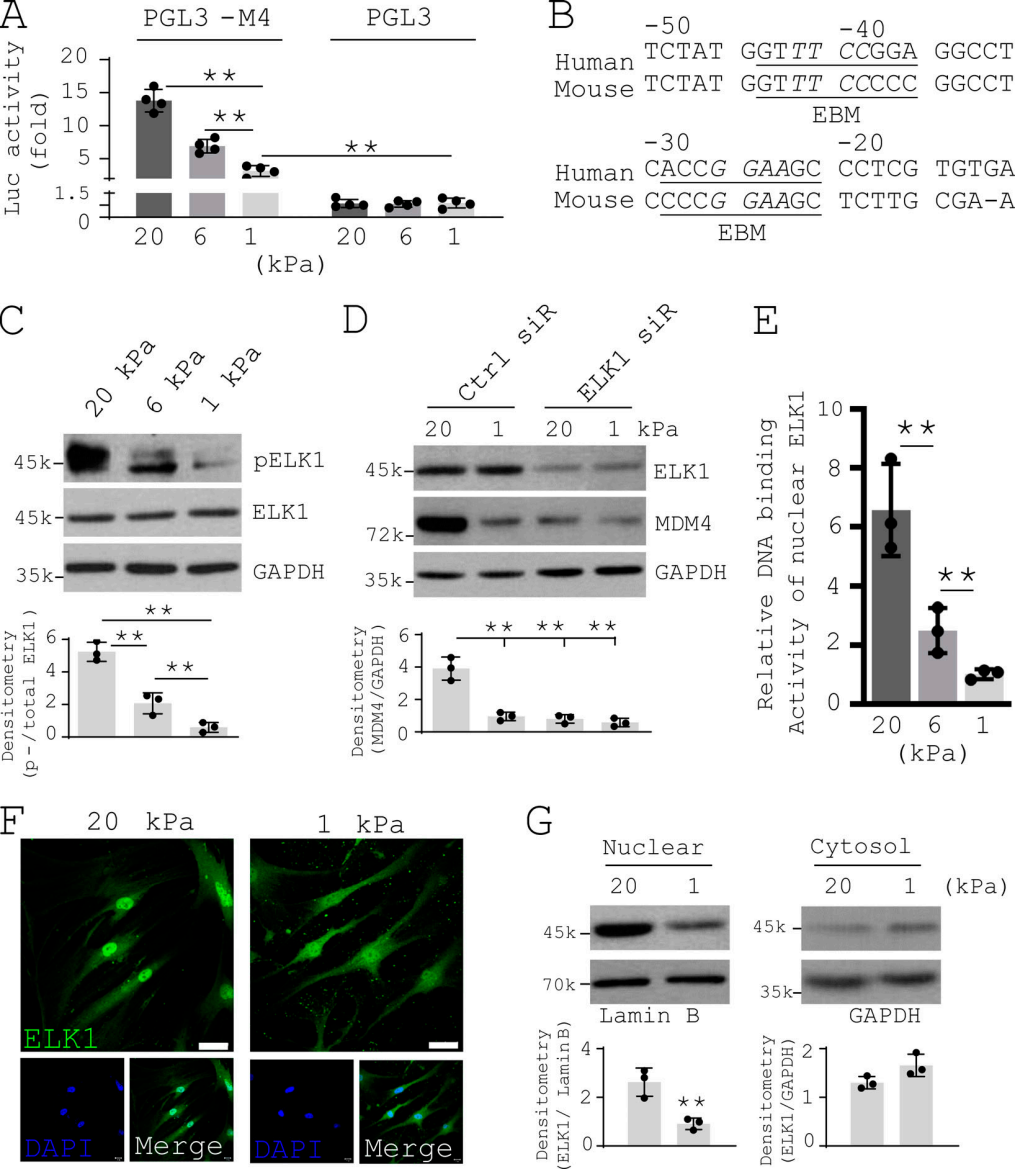
**ELK1 mediates matrix stiffness–regulated MDM4 expression. (A)** The 1-kb proximal regulatory region of *MDM4* was cloned into a promoter reporter (PGL3). PGL3-M4 and the empty vector were transfected into human lung fibroblasts by electroporation. Cells were then cultured on 1–20-kPa PA gels for 48 h. Promoter activity was determined by luciferase-based assay. **(B)** Schematic depiction of two EBMs in the proximal promoter of human *MDM4* and mouse *Mdm4*. **(C)** Human lung fibroblasts were cultured on 1–20-kPa PA gels. Levels of phospho-ELK1 (pELK1) and total ELK1 were determined by immunoblot. **(D)** Human lung fibroblasts were cultured on 1- or 20-kPa PA gels in the presence of 50 nM ELK1 siRNAs (siR) or the control siRNAs. Levels of ELK1 and MDM4 were determined by immunoblot. GAPDH was used for loading control. **(E)** Nuclear extracts derived from human lung fibroblasts cultured on 1–20-kPa PA gels were incubated with immobilized oligonucleotides containing EBMs. ELK1-specific antibodies were added to the reactions followed by incubation with a secondary HRP-conjugated antibody. EBM binding activity was quantified by colorimetric ELISA. **(F)** IF analysis of ELK1 subcellular localization in human lung fibroblasts cultured on 1- or 20-kPa PA gels. Nuclei were stained by DAPI. Scale bars = 50 µm. **(G)** Subcellular fractionation and immunoblot analysis of ELK1 localization in human lung fibroblasts cultured on 1- or 20-kPa PA gels. GAPDH was used as loading control for cytoplasmic protein and lamin B as loading control for nuclear protein. Results are the means ± SD of three or four separate experiments. **, P < 0.01 (ANOVA). k, kilodalton.

To determine the mechanisms by which stiff matrix up-regulates MDM4 expression, we cloned the 5′ regulatory region (−933 nt to +80 nt) of human *MDM4*. Luciferase-based promoter activity assays showed that this 1-kb proximal DNA sequence had DNA promoter activity, and the promoter activity was increased by matrix stiffening (Fig. 3 A). Bioinformatics analysis revealed that the proximal promoter region, which is highly conserved between human and mice, harbors ELK1 transcription factor–binding motifs (EBMs; Fig. 3 B; Dalton and Treisman, 1992; Macleod et al., 1992). It has been shown that ELK1 transactivation activity is regulated by phosphorylation at residue Ser383 (Besnard et al., 2011). We found that human lung fibroblasts cultured on stiffer matrix expressed higher levels of phospho-ELK1 (Ser383) than cells cultured on softer matrix (Fig. 3 C). Knockdown of ELK1 by siRNAs blocked stiff matrix–dependent MDM4 expression (Fig. 3 D). Furthermore, matrix stiffening increased nuclear ELK1 binding to immobilized oligonucleotides containing EBMs (Fig. 3 E). Confocal immunofluorescent (IF) microscopy and subcellular fractionation analysis demonstrated that stiff matrix increased nuclear translocation of ELK1 compared with soft matrix (Fig. 3, F and G). Taken together, these data suggest that transcription factor ELK1 mediates stiff matrix–induced MDM4 expression.

### Reducing matrix stiffness sensitizes lung myofibroblasts to apoptosis by induction of MDM4**–**p53-dependent Fas expression

**Figure 4.**
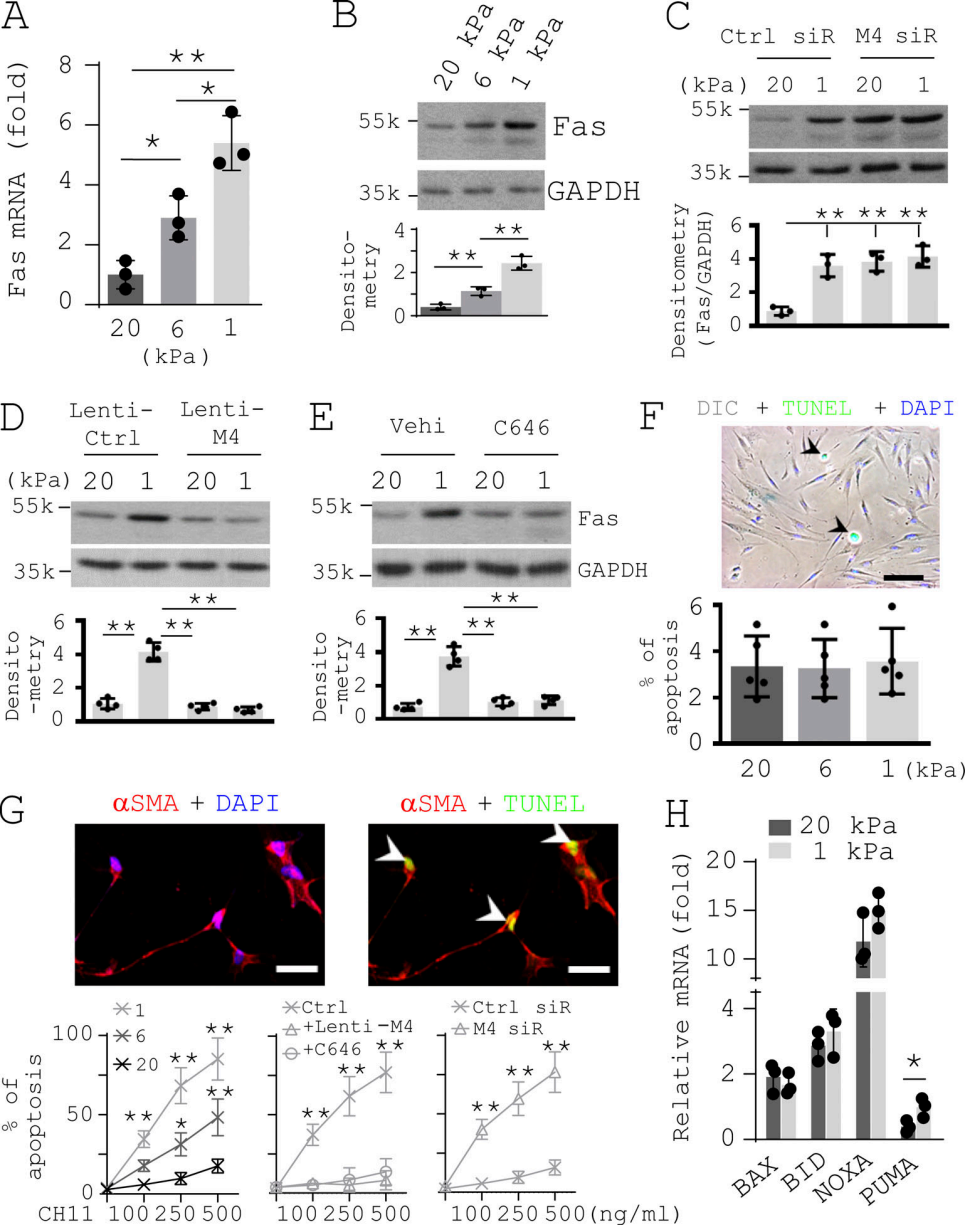
**Reducing matrix stiffness sensitizes lung myofibroblasts to apoptosis by promoting MDM4–p53-dependent Fas expression. (A)** Human IPF lung myofibroblasts were cultured on 1–20-kPa PA gels. Levels of Fas mRNA were determined by qPCR. **(B) **Levels of Fas protein were determined by immunoblot. **(C–E)** IPF lung myofibroblasts were cultured on 1- or 20-kPa PA gels in the presence of 50 nM MDM4 siRNA or the control siRNA (C), MDM4-expressing lentiviral vector or the control lentiviral vector (D), and 25 µM C646 or the vehicle (Vehi) control (E). Levels of Fas protein were determined by immunoblot. **(F)** IPF lung myofibroblasts were cultured on 1–20-kPa PA gels. Cell apoptosis was evaluated by TUNEL staining and IF/differential interference contrast (DIC) microscopy. Nuclei were stained by DAPI. A representative image shows cell apoptosis (arrowheads) under 20-kPa gel conditions. **(G)** IPF lung myofibroblasts were cultured on 1–20-kPa PA gels in the presence or absence of CH11 (100–500 ng/ml), MDM4-expressing lentiviruses or the control lentiviruses, C646, and MDM4 siRNA (siR) or the control siRNA. Cell apoptosis was evaluated by TUNEL staining and confocal IF microscopy. Nuclei were stained by DAPI. Representative images showed cell apoptosis (arrowheads) under 1-kPa gel conditions in the presence of 250 ng/ml CH11. **(H)** qPCR analysis for transcription of BAX, BID, NOXA, and PUMA in IPF lung myofibroblasts cultured on 1- and 20-kPa PA gels. GAPDH was used as internal reference control in qPCR analysis and as loading control in immunoblot analysis. Results are the means ± SD of three to five separate experiments. Apoptosis was evaluated in at least 200 lung myofibroblasts per experiment. Scale bar = 250 µm (F) and 50 µm (G). *, P < 0.05; **, P < 0.01 (ANOVA). k, kilodalton.

It has been reported that human embryonic lung fibroblast cell line (IMR-90) cultured on stiff matrix expressed a reduced level of p53-regulated death receptor Fas compared with cells cultured on soft matrix (Dodi et al., 2018). Consistent with this finding, we found that reducing matrix stiffness up-regulated Fas expression at both the mRNA and protein level in primary lung myofibroblasts isolated from patients with IPF (Fig. 4, A and B). Furthermore, knockdown of MDM4 by siRNAs resulted in increased Fas expression on stiff matrix, whereas overexpression of MDM4 by lentiviral vectors or repression of p300-dependent p53 acetylation/activation by C646 inhibited Fas expression on soft matrix (Fig. 4, C–E). These findings suggest that matrix stiffness regulates Fas expression in IPF lung myofibroblasts by a MDM4–p53-dependent mechanism.

We observed minimal apoptosis when IPF lung myofibroblasts were cultured under either soft or stiff matrix conditions (Fig. 4 F). However, when cells were incubated with increasing concentrations of anti-Fas antibody (a Fas ligand [FasL]), we observed dose-dependent cell apoptosis, which was more prominent under softer matrix conditions than under stiffer matrix conditions (Fig. 4 G). Overexpression of MDM4 or repression of p53 activity blocked FasL-induced lung myofibroblast apoptosis under soft matrix conditions, whereas inhibition of MDM4 expression promoted lung myofibroblast apoptosis under stiff matrix conditions (Fig. 4 G). It is known that p53 targets transcriptional expression of the components of both the extrinsic and intrinsic apoptotic pathways (Haupt et al., 2003). The intrinsic apoptotic pathway is dominated by the Bcl-2 family of proteins. A key subset of the Bcl-2 family genes, including *BAX*, *NOXA*, *PUMA*, and *BID*, are p53 targets (Aubrey et al., 2018). We found that IPF lung myofibroblasts expressed higher levels of PUMA at the mRNA level on soft matrix versus stiff matrix, whereas mRNA expression of BAX, NOXA, and BID were equivalent under different matrix stiffness conditions (Fig. 4 H). Since IPF lung myofibroblasts cultured on soft matrix did not undergo significant apoptosis (Fig. 4 F), soft matrix–induced transcription of PUMA may not be sufficient to induce myofibroblast apoptosis, presumably due to a relatively lower of PUMA at baseline in lung myofibroblasts. Together, these findings suggest that reducing matrix stiffness sensitizes lung myofibroblasts to apoptosis by induction of MDM4–p53-dependent Fas expression.

### Reducing matrix stiffness facilitates macrophage phagocytosis of apoptotic lung myofibroblasts by induction of MDM4**–**p53-dependent expression of death domain 1α (DD1α) on the cell surface of lung myofibroblasts

**Figure 5.**
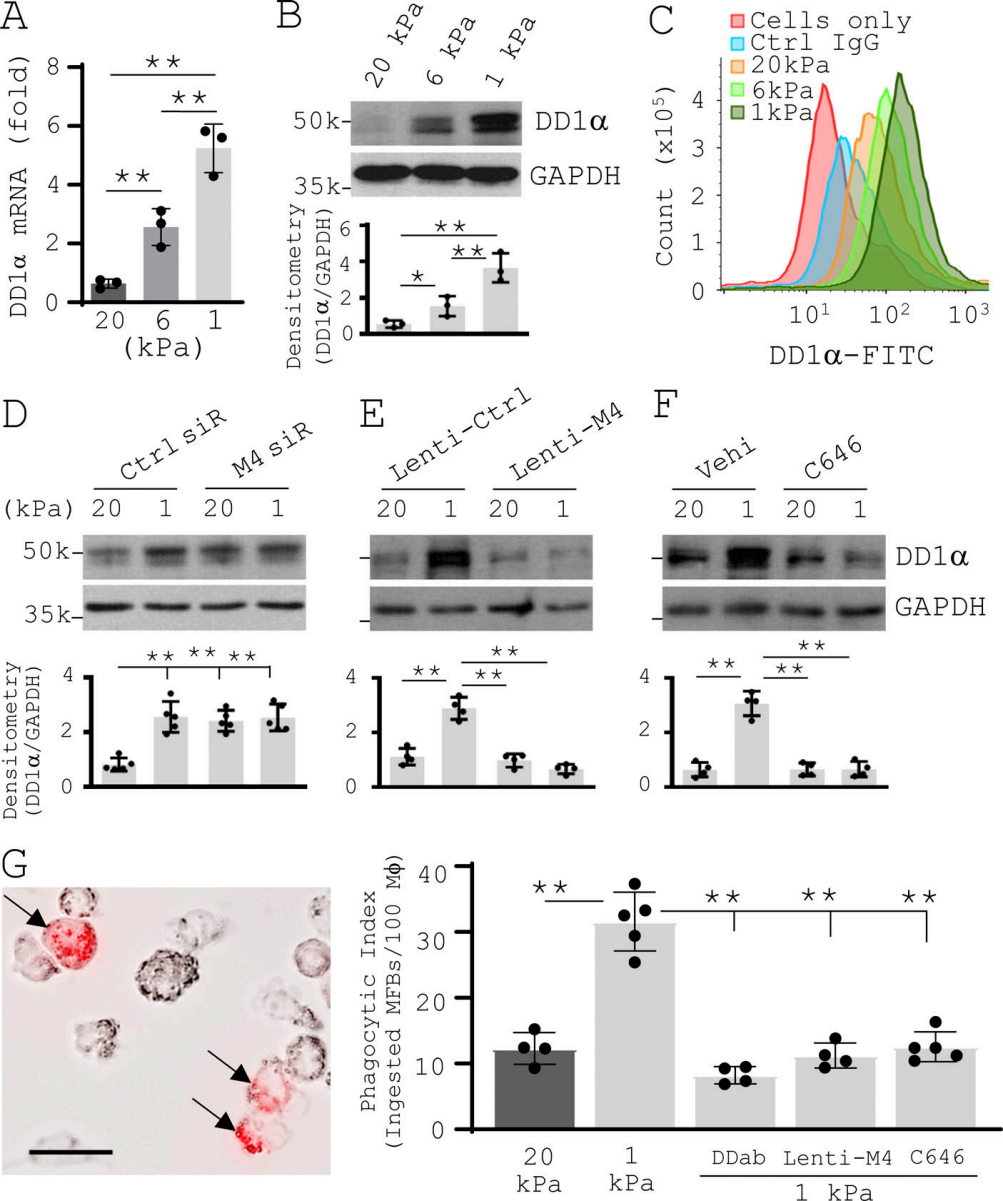
**Reducing matrix stiffness promotes MDM4–p53-dependent DD1α expression by lung myofibroblasts, facilitating macrophage phagocytosis of apoptotic lung myofibroblasts. (A)** Human IPF lung myofibroblasts were cultured on 1–20-kPa PA gels. Levels of DD1α mRNA were determined by qPCR. **(B)** Levels of DD1α protein were determined by immunoblot. **(C)** Levels of DD1α expression on the cell surface were evaluated by flow cytometry. Cells incubated with nonimmune control IgG and cells alone were included as negative controls. **(D–F)** IPF lung myofibroblasts were cultured on 1- or 20-kPa PA gels in the presence of 50 nM MDM4 siRNA (siR) or the control siRNA (D), MDM4-expressing lentiviral vector or the control lentiviral vector (E), and 25 µM C646 or the vehicle (Vehi) control (F). Levels of DD1α protein were determined by immunoblot. **(G)** IPF lung myofibroblasts were cultured on 1- or 20-kPa PA gels in the presence or absence of 10 µg/ml DD1a neutralizing antibody (DDab), MDM4-expressing lentiviral vector, or 25 µM C646. After 24 h, cells were treated with 500 ng/ml CH11 to induce apoptosis. Apoptotic cells were collected by flow cytometry cell sorting, labeled by pHrodo Red, and incubated with THP1 at 1:10 to 1:15 ratio (THP1/myofibroblasts). Phagocytosis was observed were under a fluorescence microscope using bright field or Texas Red filter set. The phagocytic index was calculated as described in Materials and methods. A representative image shows macrophage phagocytosis (arrowheads) of pHrodo Red–labeled apoptotic IPF lung myofibroblasts derived from 1-kPa gel conditions. GAPDH was used as internal reference control in qPCR analysis and as loading control in immunoblot analysis. Results are the means ± SD of three to five separate experiments. Phagocytosis was evaluated in at least 500 macrophages per experiment. Scale bar = 20 µm. *, P < 0.05; **, P < 0.01 (ANOVA). k, kilodalton.

DD1α is a p53-targeted engulfment receptor that mediates the interactions between macrophages and apoptotic cells (Yoon et al., 2015). We found that reducing matrix stiffness increased DD1α mRNA expression and protein expression, both in the cytosol and on the cell surface, in IPF lung myofibroblasts (Fig. 5, A–C). Knockdown of MDM4 by siRNAs promoted DD1α expression on stiff matrix, whereas lentiviral vector–mediated MDM4 overexpression or pharmacological suppression of p53 activity inhibited DD1α expression on soft matrix (Fig. 5, D–F). These findings suggest that MDM4-dependent p53 activation mediates soft matrix–induced DD1α expression. To investigate the effect of matrix stiffness–regulated DD1α expression on the clearance of apoptotic lung myofibroblasts by macrophages, we treated lung myofibroblasts cultured on stiff or soft matrix substrates with FasL to induce apoptosis. An equal number of apoptotic cells were labeled by pHrodo Red and then incubated with macrophages (THP1). Phagocytosis assays showed that apoptotic lung myofibroblasts derived from soft matrix had a higher phagocytic index than apoptotic cells derived from stiff matrix (Fig. 5 G). This result suggests that soft microenvironment promotes the clearance of apoptotic lung myofibroblasts by macrophages. DD1α blocking antibody or pretreatment of lung myofibroblasts with lentiviral vectors expressing exogenous MDM4 or with C646 inhibited the engulfment of apoptotic lung myofibroblasts by macrophages under soft matrix conditions (Fig. 5 G). These data suggest that reducing matrix stiffness facilitates the clearance of apoptotic lung myofibroblasts by macrophages through MDM4–p53-dependent DD1α expression.

### Reducing matrix stiffness promotes macrophage chemotaxis by a MDM4**–**p53-dependent CX3CL1 paracrine signal derived from lung myofibroblasts

**Figure 6.**
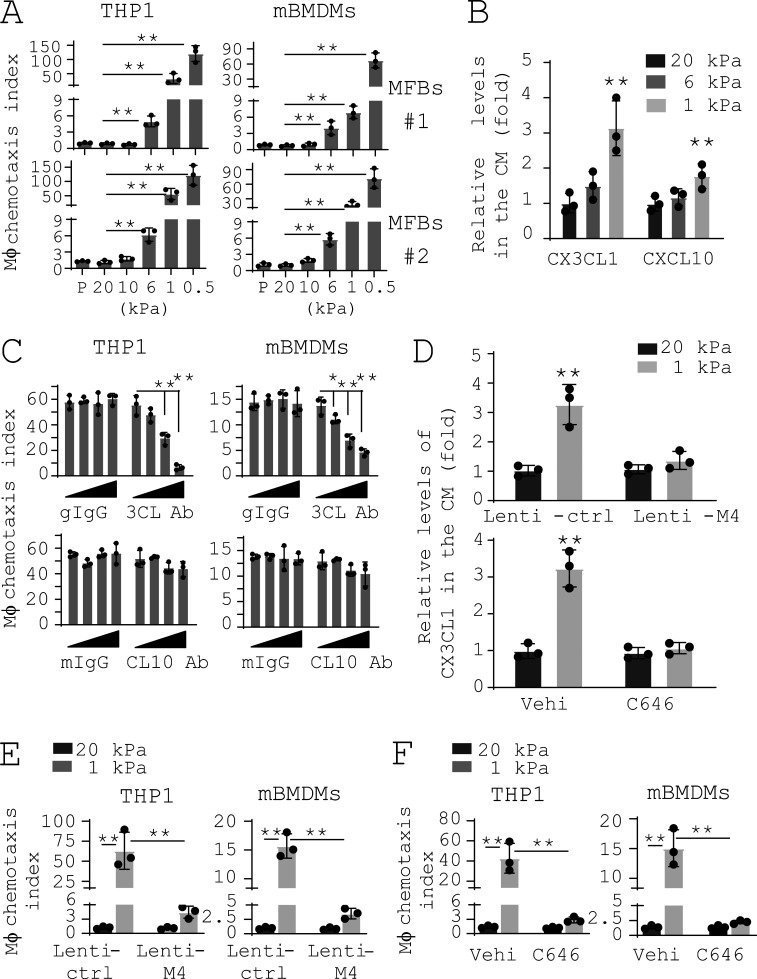
**Reducing matrix stiffness promotes macrophage chemotaxis by a MDM4–p53-dependent CX3CL1 paracrine signal derived from lung myofibroblasts. (A)** An equal number of IPF lung myofibroblasts were cultured on 0.5–20-kPa PA gels or plastic surface (P). CM was collected and incubated with 2 × 10^5^ THP1 or mBMDMs in 96-Transwell plates. Migrating macrophages were stained by calcein-AM. Chemotaxis index was calculated as a ratio of quantitative fluorescence of cells incubated with the CM to cells incubated with plain DMEM. Mϕ, macrophage. **(B)** Relative levels of CX3CL1 and CXCL10 in the CM collected from IPF lung myofibroblasts cultured on 1–20-kPa PA gels were determined by ELISA. **(C)** IPF lung myofibroblasts were cultured on 1-kPa PA gels. CM were collected and incubated with THP1 or mBMDMs in the presence of increasing concentrations (0, 0.1, 1, and 10 µg/ml) of CX3CL1 neutralizing antibody (3CL Ab), nonimmune goat IgG (gIgG), CXCL10 neutralizing Ab (CL10 Ab), or mouse IgG (mIgG). Macrophage chemotaxis index was determined as described above. **(D)** IPF lung myofibroblasts were cultured on 1- or 20-kPa PA gels in the presence or absence of MDM4-expressing lentiviral vector or 25 µM C646. Levels of CX3CL1 in the CM were determined by ELISA. **(E and F)** CM collected from cells treated in D were incubated with THP1 or mBMDMs. Effects of MDM4 overexpression (E) and C646 treatment (F) on macrophage chemotaxis were determined as described above. Results are the means ± SD of three separate experiments. *, P < 0.05; **, P < 0.01 (ANOVA).

**Figure S3.**
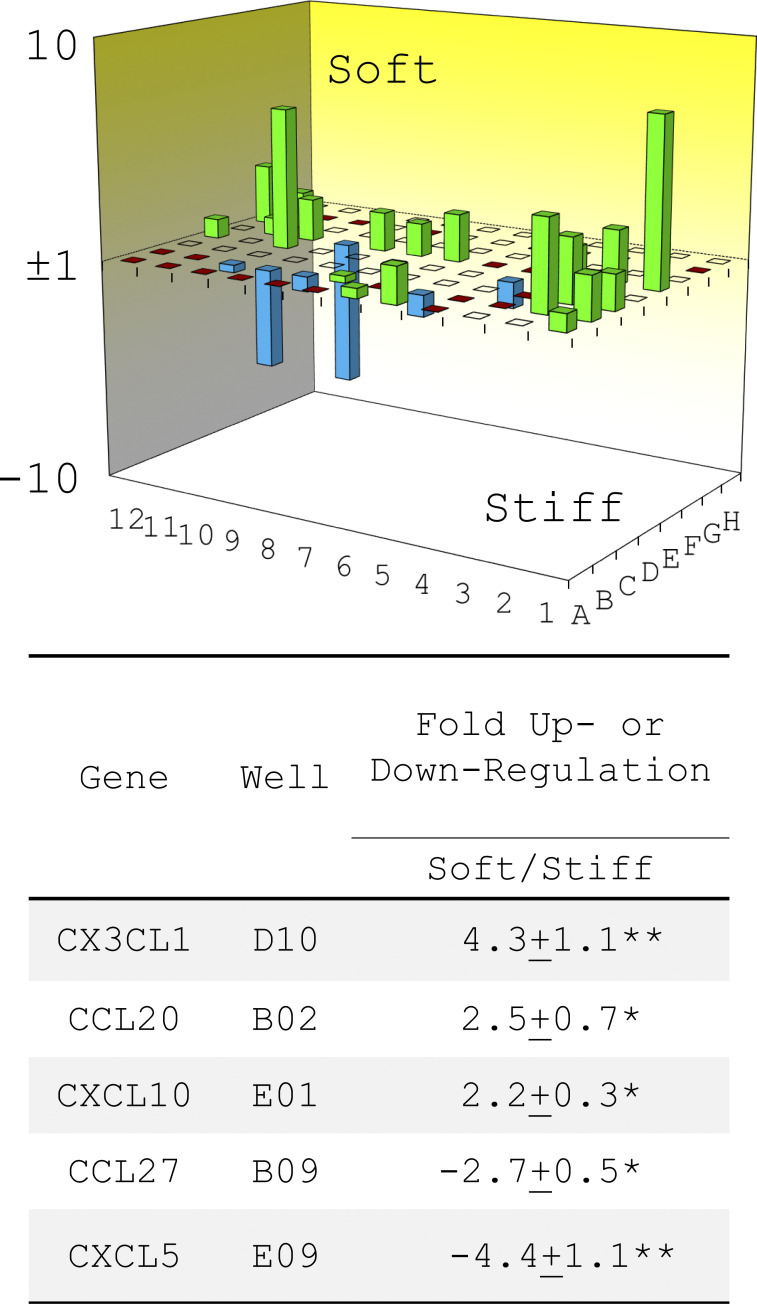
**Effects of matrix stiffness on transcription of chemokines in primary lung myofibroblasts isolated from patients with IPF.** Relative transcriptional levels of 84 chemokines and receptors were determined by RT^2^ PCR array (array code PAHS-022Z; Qiagen). Up- or down-regulation of five chemokines on soft versus stiff matrix is shown. Results are the means ± SD of three separate experiments, each performed in triplicate. *, P < 0.05; **, P < 0.01 (ANOVA).

To evaluate the potential effect of lung myofibroblasts cultured under varying matrix stiffness on macrophage migration, we collected conditioned medium (CM) from two independent IPF lung myofibroblast populations cultured on 0.5–20-kPa PA gels and incubated the CM with both human THP1 macrophages and mouse bone marrow–derived macrophages (mBMDMs) in Transwell. Cell migration assays showed that the CM collected from lung myofibroblasts on softer matrix attracted more macrophages than the CM collected from cells on stiffer matrix (Fig. 6 A). This finding suggests that reducing matrix stiffness indirectly promotes macrophage migration, presumably by a paracrine signal(s) derived from lung myofibroblasts. Using RT-PCR–based array analysis, we identified a group of chemokines whose transcriptions were differentially regulated by matrix stiffness in lung myofibroblasts (Fig. S3). ELISA confirmed that protein levels of CX3CL1 and CXCL10, both of which are chemoattractants of macrophages (Corbera-Bellalta et al., 2016; Gevrey et al., 2005; Truman et al., 2008), were increased under soft matrix conditions compared with stiff matrix conditions (Fig. 6 B). Pretreatment of the CM with CX3CL1 neutralizing antibody dose-dependently inhibited macrophage chemotaxis, whereas pretreatment of CXCL10 neutralizing Ab had no significant effects (Fig. 6 C). Lentiviral vector–mediated overexpression of MDM4 or repression of p300-dependent p53 acetylation/activation by C646 blocked matrix stiffness-dependent CX3CL1 expression (Fig. 6 D) as well as the paracrine effect of lung myofibroblasts on macrophage chemotaxis (Fig. 6, E and F). These data suggest that reducing matrix stiffness promotes MDM4–p53-dependent CX3CL1 expression by lung myofibroblasts which acts as a paracrine signal to recruit macrophages.

### Nonenzymatic advanced glycation end product (AGE) cross-linking is increased in human IPF and bleomycin injury-induced lung fibrosis in aged mice

**Figure S4.**
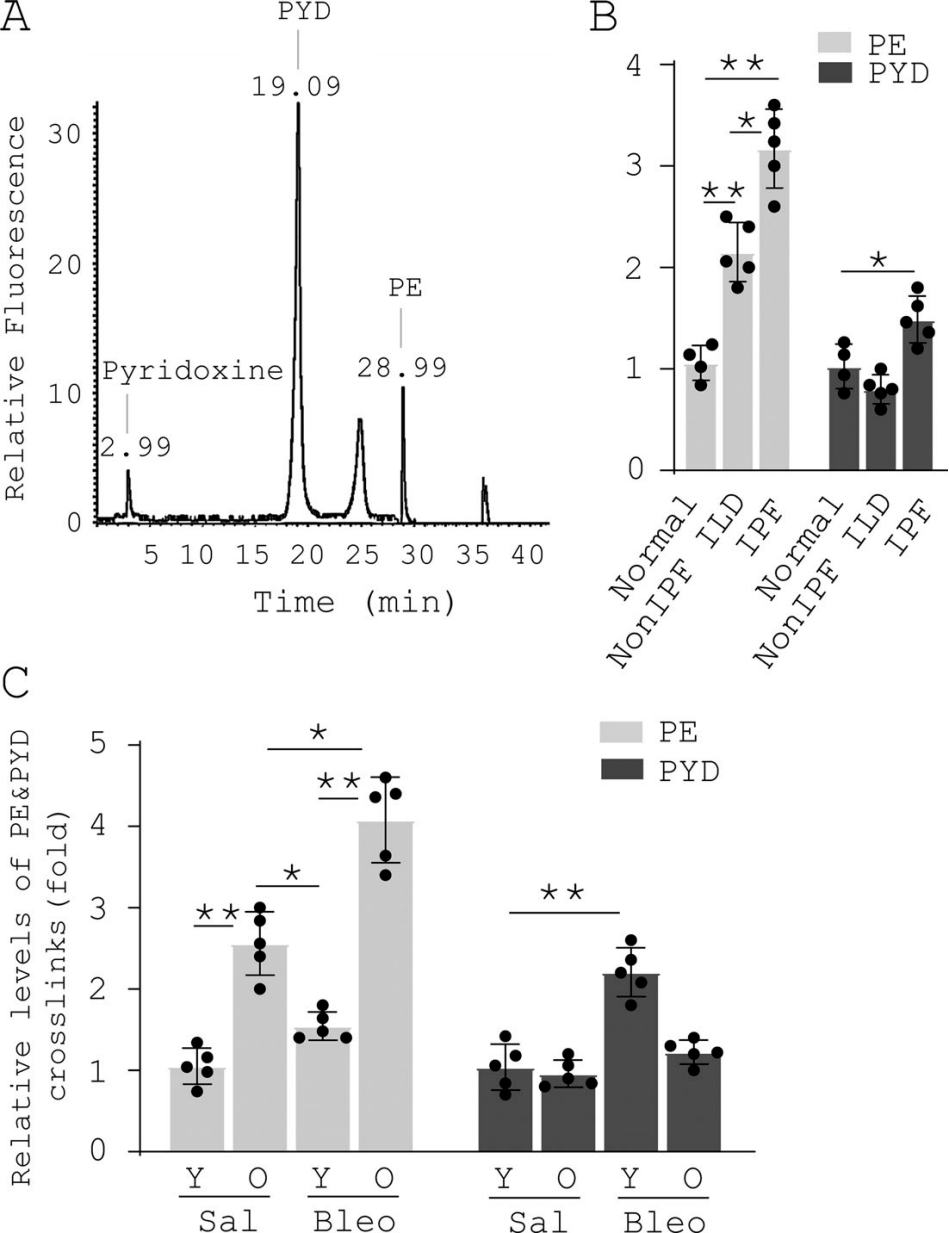
**Nonenzymatic AGE cross-linking is increased in human IPF and experimental lung fibrosis in aged mice. (A)** Evaluation of nonenzymatic PE cross-linking and enzymatic PYD cross-linking by single reverse-phase HPLC-fluorescence detection. **(B)** Relative levels of PE and PYD in normal (*n* = 4), non-IPF ILD (*n* = 5), and IPF (*n* = 5) lungs. **(C)** Relative levels of PE and PYD in the lungs of saline- (Sal) or bleomycin- (Bleo) treated young (Y) and old (O) mice (*n* = 5 per group). Results are the means ± SD of three separate experiments, each performed in triplicate. *, P < 0.05; **, P < 0.01 (ANOVA).

Aging tissues are characterized by an increase in the formation of AGEs (Simm et al., 2015). The ECM, in particular collagen matrix, is highly sensitive to glycation due to a slow turnover rate (Verzijl et al., 2000). AGEs drive nonenzymatic cross-linking of the ECM, thereby contributing to matrix stiffening (Furber, 2006; Simm, 2013; Verzijl et al., 2002; Wells, 2008). To evaluate nonenzymatic AGE cross-linking in normal versus fibrotic lungs, we measured pentosidine (PE), a commonly used marker of glycation cross-linking (Sell and Monnier, 1989), by single reversed-phase high-performance liquid chromatography (HPLC; Fig. S4 A). We also measured lysyl oxidase–mediated hydroxylysylpyridinoline (PYD) cross-linking (van der Slot et al., 2004) for evaluation of enzymatic cross-linking. We found that nonenzymatic PE was increased in human IPF lungs (66–74 yr) and non-IPF interstitial lung disease (ILD) lungs (64–75 yr) compared with normal donor lungs (32–45 yr; Fig. S4 B). IPF lungs had greater PE cross-linking than non-IPF ILD lungs. PYD cross-linking was increased in IPF lungs compared with non-IPF ILD lungs and normal donor lungs (Fig. S4 B). Mouse studies showed that in the saline-treated control group, aged mice (15 mo) had more PE in the lung than young mice (2 mo; Fig. S4 C). Bleomycin treatment increased PE in both young and aged mice compared with saline-treated counterparts. PYD cross-links were found to be equivalent between young and aged mice in the saline group (Fig. S4 C). Bleomycin treatment significantly increased PYD cross-links in young mice and slightly increased PYD cross-links (statistically insignificant) in aged mice. Collectively, these data suggest that nonenzymatic AGE cross-linking increases in aged lungs and accelerates in lung fibrosis.

### Destiffening of the fibrotic lungs by targeting AGE cross-linking promotes lung fibrosis resolution in aged mice

**Figure 7.**
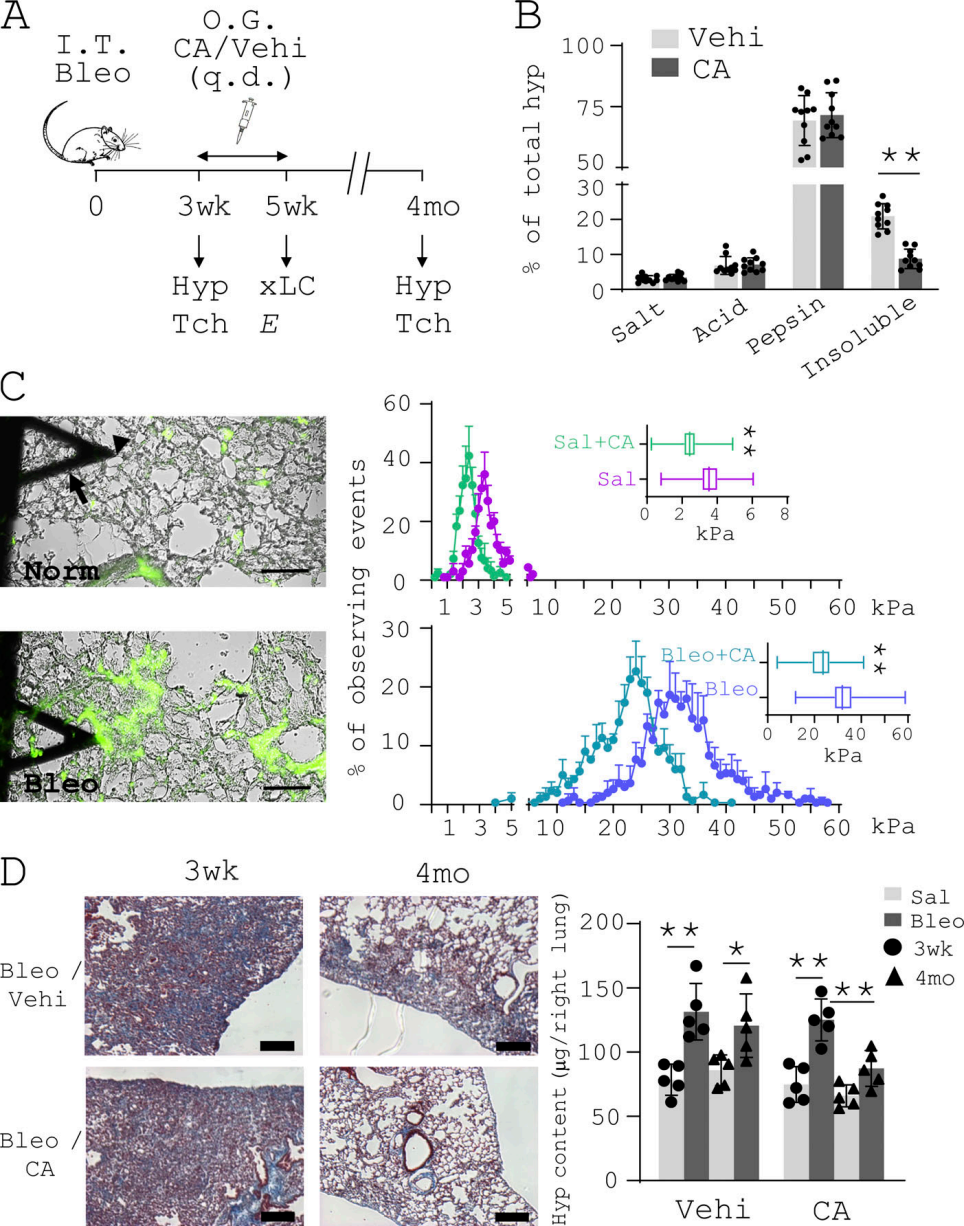
**Inhibition of AGE cross-linking destiffens the fibrotic lungs and promotes lung fibrosis resolution in aged mice. (A)** Schematic depiction of the course of animal experiments. *E*, elastic moduli (Young’s moduli) of mouse lung tissues; Hyp, Hyp content assay; I.T. Bleo, intratracheal bleomycin; O.G., oral gavage; q.d., *quaque die*; Tch, trichrome staining; Vehi, vehicle; xLC, collagen cross-linking assay. **(B)** Collagen cross-linking in CA- and vehicle (DMSO)–treated fibrotic mouse lungs (*n* = 10 per group) was evaluated by stepwise collagen solubilization assay. Lung tissues were serially extracted by neutral salt buffer (Salt), acetic acid (Acid), and acid pepsin (Pepsin). The remaining insoluble fraction contains highly cross-linked collagens. Collagens in each fraction were quantified by Hyp assay and expressed as percentage of total collagens. **(C)** Mechanical testing of unfixed native mouse lung tissues was performed by AFM microindentation. Tissue sections were stained by a collagen-Col-F collagen-binding reagent to facilitate localization of specific lung areas (e.g., the alveolar wall and collagen-enriched fibrotic area) and positioning of the cantilever (arrow) tip (arrowhead). Bar graphs show the distribution of lung stiffness in normal (Norm) and bleomycin-treated (Bleo) aged mice after CA or vehicle treatments (*n* = 5 per group). Scale bars = 100 µm. Sal, saline. **(D)** Representative images show trichrome staining of collagens in paraffin-embedded fibrotic lung tissue harvested from CA- or vehicle-treated mice at 3 wk and 4 mo. Lung fibrosis resolution was assessed by decreases in lung Hyp contents comparing 3 wk to 4 mo in the bleomycin-treated group versus the control (saline) group (*n* = 8 per group). Results are the means ± SD from 5–10 mice per treatment group, each performed in triplicate. Scale bars = 150 µm. *, P < 0.05; **, P < 0.01 (ANOVA).

Chebulic acid (CA) is a potent breaker and inhibitor of AGE cross-linking (Aldini et al., 2013; Lee et al., 2007; Lee et al., 2010; Lee et al., 2014; Nagai et al., 2012; Pellati et al., 2013). It has been used to treat experimental diabetic mellitus in rats (Kim et al., 2011; Kim et al., 2012a; Pellati et al., 2013; Silawat and Gupta, 2013). In this study, we tested the potential of CA to destiffen the fibrotic lungs and to reverse persistent lung fibrosis in aged mice (Fig. 7 A). Treatment of aged mice by CA for 14 d consecutively at 3 wk after bleomycin administration significantly decreased the proportion of insoluble (cross-linked) collagen in the fibrotic lungs of aged mice, as demonstrated by a stepwise collagen solubilization assay (Fig. 7 B). Atomic force microscopy (AFM)–mediated mechanical testing of unfixed native lungs demonstrated a decrease in the overall elastic moduli (*E*) in both normal and fibrotic mice after CA treatment (Fig. 7 C). Hydroxyproline (Hyp) content assay showed that the amount of Hyp in CA-treated mouse lungs decreased from 145 ± 13 µg at 3 wk to 111 ± 16 µg at 4 mo (ΔHyp = 34 ± 13 µg), whereas the amount of Hyp in the control mouse lungs decreased from 153 ± 22 µg to 145 ± 17 µg (ΔHyp = 8 ± 7 µg) in this period of time (Fig. 7 D). Masson’s trichrome staining showed greater reduction in collagen staining and larger improvement of lung morphology in CA-treated mice compared with vehicle-treated control mice (Fig. 7 D). Together, these data suggest that destiffening of the fibrotic ECM by targeting nonenzymatic AGE cross-linking promotes lung fibrosis resolution in aged mice.

### Matrix destiffening activates the MDM4–p53 pathway and promotes the clearance of myofibroblasts in the fibrotic lungs of aged mice

**Figure 8.**
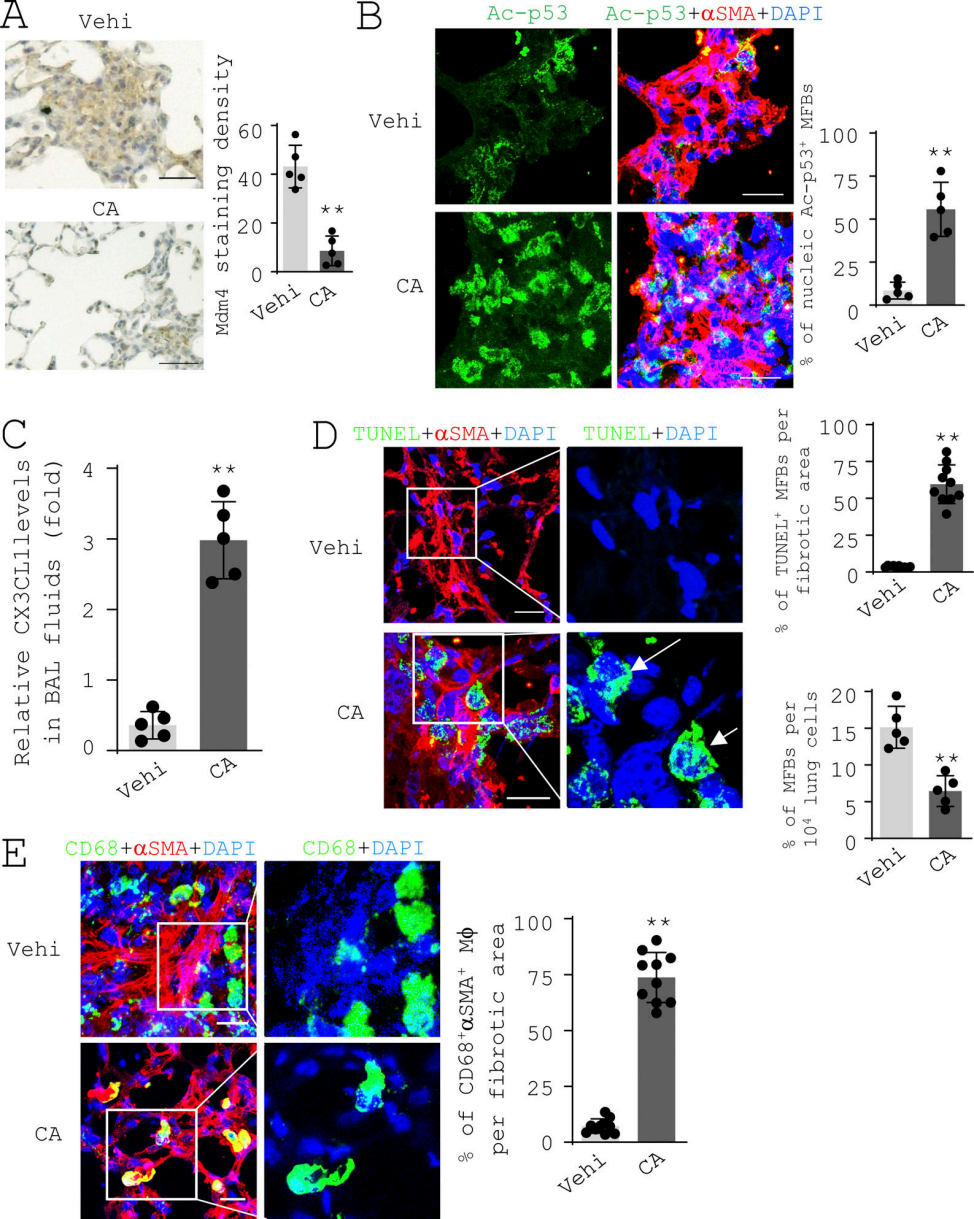
**Matrix destiffening activates the MDM4–p53 pathway and promotes the clearance of lung myofibroblasts by macrophages in the fibrotic lungs of aged mice. (A)** IHC analysis of Mdm4 expression in aged fibrotic mice treated by CA or vehicle at 4 mo (*n* = 5 per group). Mdm4-positive signals were quantified by ImageJ. Scale bars = 50 µm. **(B)** Confocal IF microscopy to evaluate Ac-p53 expression in αSMA-expressing myofibroblasts in the fibrotic lungs of aged mice treated with CA or vehicle. Scale bars = 50 µm. **(C)** Levels of CX3CL1 in the BAL fluids of aged fibrotic mice treated with CA or vehicle were quantified by ELISA (*n* = 5 per group). **(D)** TUNEL staining and confocal IF microscopy to evaluate lung myofibroblast apoptosis in the fibrotic lungs of aged mice treated with CA or vehicle. Scale bars = 50 µm. **(E)** Confocal IF microscopy to evaluate macrophage (CD68) engulfment of myofibroblasts (αSMA) in the fibrotic lungs of aged mice treated with CA or vehicle. Scale bars = 50 µm. Quantitative IF analysis was performed in 5–10 randomly selected lung areas. Results are the means ± SD from a total of five mice per treatment group, each performed in triplicate. Nuclei were stained with DAPI. **, P < 0.01 (ANOVA).

**Figure S5.**
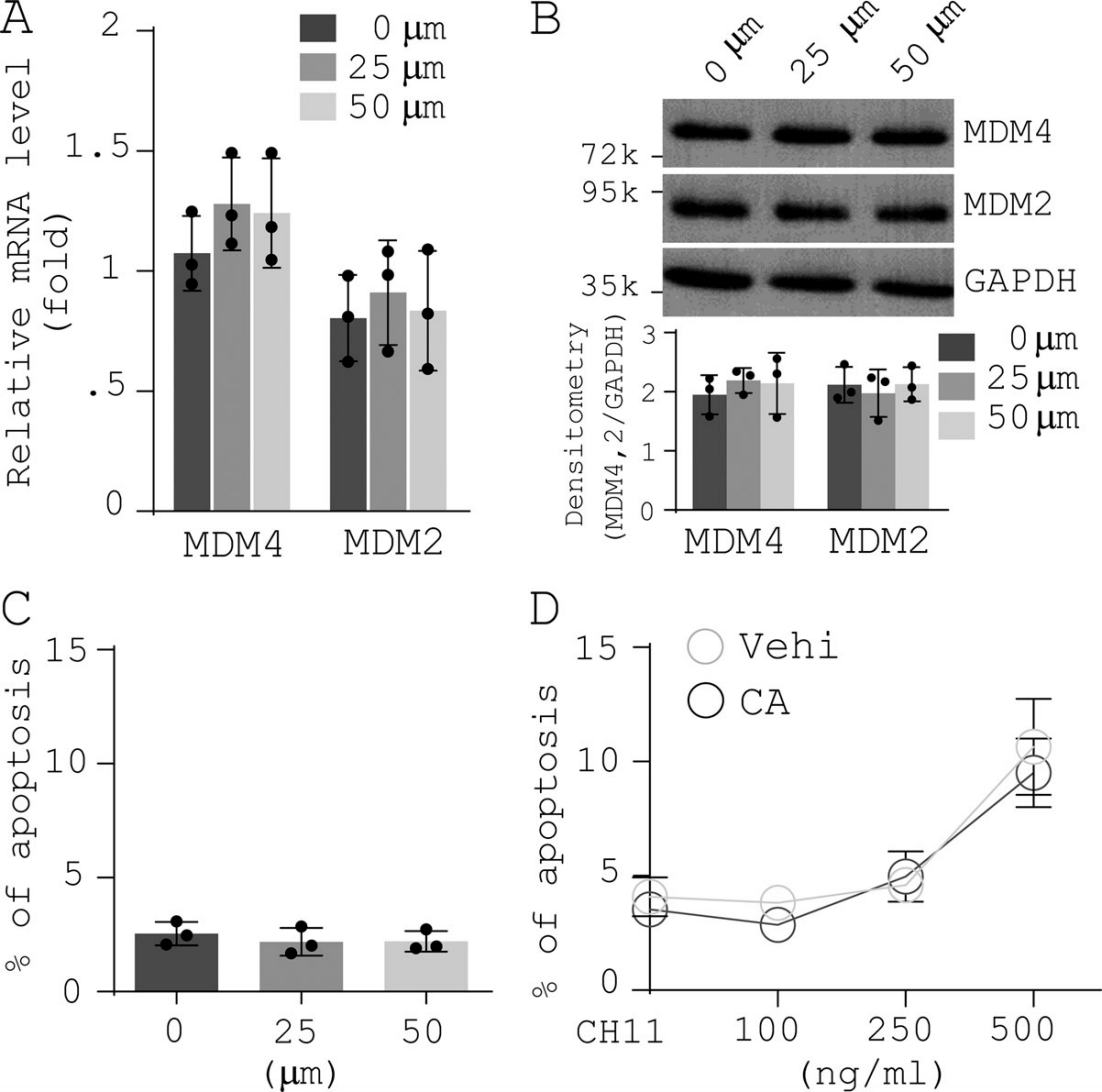
**Effects of CA on MDM4 and MDM2 expression, lung myofibroblast apoptosis, and sensitization of lung myofibroblasts to FasL-induced apoptosis.** Human IPF lung myofibroblasts were treated by increasing concentrations of CA.** (A)** Levels of MDM4 and MDM2 mRNA were determined by qPCR.** (B)** Levels of MDM4 and MDM2 protein were determined by immunoblot. **(C)** Apoptosis was evaluated by TUNEL staining and IF/differential interference contrast (DIC) microscopy as described in Fig. 4 F. **(D)** IPF lung myofibroblasts were pretreated by 50 µm CA followed by incubation with increasing concentrations of CH11. Cell apoptosis was evaluated by TUNEL staining and confocal IF microscopy as described in Fig. 4 G. GAPDH was used as internal reference control in qPCR analysis and as loading control in immunoblot analysis. Results are the means ± SD of three separate experiments, each performed in triplicate. Vehi, vehicle. k, kilodalton.

IHC analysis showed that aged mice treated by CA had weaker Mdm4-positive signals in the fibrotic lung area compared with the control mice (Fig. 8 A). Costaining of acetylated p53 and αSMA by IF showed that CA treatment significantly increased acetylated p53 in the nuclei of αSMA-expressing lung myofibroblasts (Fig. 8 B). CA treatment increased the level of CX3CL1 in the bronchoalveolar lavage (BAL) fluids of aged fibrotic mice, as assessed by ELISA (Fig. 8 C). CA-treated mice displayed terminal deoxynucleotidyl transferase dUTP nick end labeling (TUNEL)–positive nuclei in αSMA-expressing myofibroblasts and reduction in the percentage of myofibroblasts in the lung (Fig. 8 D), indicative of lung myofibroblasts that were undergoing apoptosis. In contrast, few apoptotic lung myofibroblasts were observed in the control mice. CD68-positive macrophages in the control mice were primarily found in the periphery of the fibrotic lesions (Fig. 8 E, top panels). In CA-treated mice, macrophages were found within the fibrotic lesions where αSMA-positive myofibroblasts were predominant (Fig. 8 E, bottom panels), implicating the clearance of myofibroblasts by macrophages. In vitro studies showed that CA treatment neither altered expression of MDM4 and MDM2 nor promoted apoptosis of lung myofibroblasts (Fig. S5), suggesting that CA does not directly attenuate Mdm4 expression or induce lung myofibroblast apoptosis. Collectively, these data provide in vivo evidence that matrix destiffening by CA treatment activates the MDM4–p53 pathway and promotes the clearance of lung myofibroblasts by macrophages in the fibrotic lungs of aged mice.

### Targeting nonenzymatic AGE cross-linking reduces IPF lung tissue stiffness and renders IPF lung collagen more susceptible to MMP1-mediated degradation ex vivo

**Figure 9.**
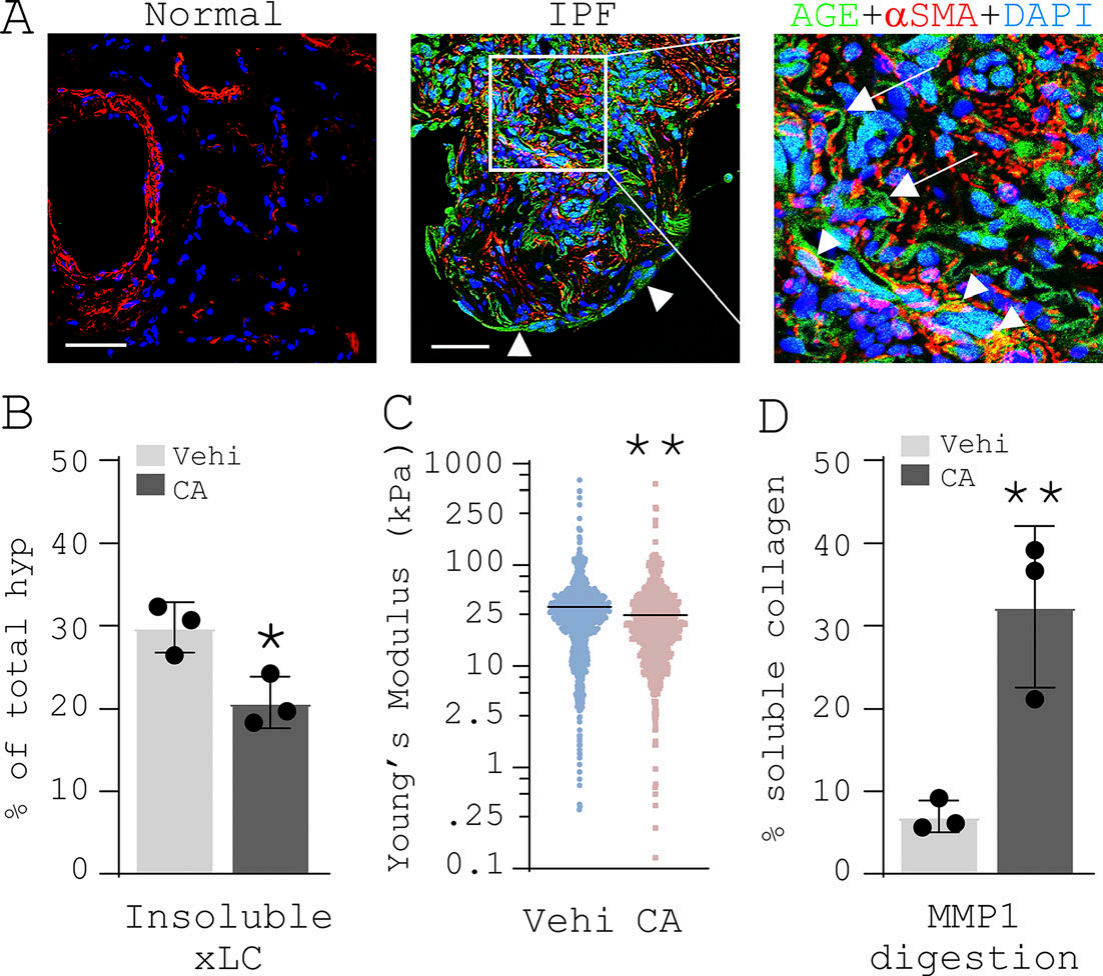
**CA treatment reduces IPF lung tissue stiffness and renders lung collagen more susceptible to MMP1-mediated degradation ex vivo. (A)** Confocal IF microscopy to analyze AGEs (green) and αSMA (red) in normal and IPF lungs (*n* = 3 per group). Nuclei were stained with DAPI. Representative images were derived from a 69-yr-old white male (IPF, past smoker) and a 32-yr-old white male (Normal, never smoker). Arrowheads in the middle panel indicate AGE-positive signals in cells lining lung epithelium. In the right panel, arrowheads indicate AGE-positive signals in αSMA-expressing myofibroblasts; arrows indicate AGE-positive signals in the ECM fibers. Scale bars = 50 µm. **(B)** Decellularized IPF lung slices were incubated with 25 µM CA or distilled DMSO (vehicle) at 37°C for 16 h. Insoluble cross-linked collagens (xLC) were quantified by stepwise collagen solubilization assay. **(C)** Tissue stiffness of decellularized IPF lungs was measured by AFM microindentation. **(D)** CA- or vehicle-treated IPF lung tissues were incubated with 10 µg/ml active MMP1 at 25°C for 2 h. Collagen degradation was evaluated by the percentage of soluble collagen in total collagens following Hyp assay. Results are mean ± SD of three IPF samples, each performed in triplicate. *, P < 0.05; **, P < 0.01 (ANOVA).

Confocal IF microscopy showed that AGE-positive signals were present in the ECM of the fibroblastic foci in IPF (Fig. 9 A, indicated by arrows in the right panel). AGE-positive signals were also found in alveolar epithelial cells (Fig. 9 A, indicated by arrowheads in the middle panel) and a subset of αSMA-expressing myofibroblasts (Fig. 9 A, yellow cells indicated by arrowheads in the right panel). Younger donor lungs had no detectable levels of AGEs (Fig. 9 A, left panel). Treatment of decellularized IPF lung tissue slices by CA reduced the proportion of cross-linked collagen ex vivo (Fig. 9 B) and decreased lung stiffness (Fig. 9 C). Furthermore, CA treatment increased the susceptibility of IPF lung tissues to MMP1 (also known as collagenase I)–mediated collagen degradation (Fig. 9 D).

### Genetic deletion of *Mdm4* in (myo)fibroblasts activates the p53-mediated fibrosis resolution pathway and reverses persistent lung fibrosis in aged mice

**Figure 10.**
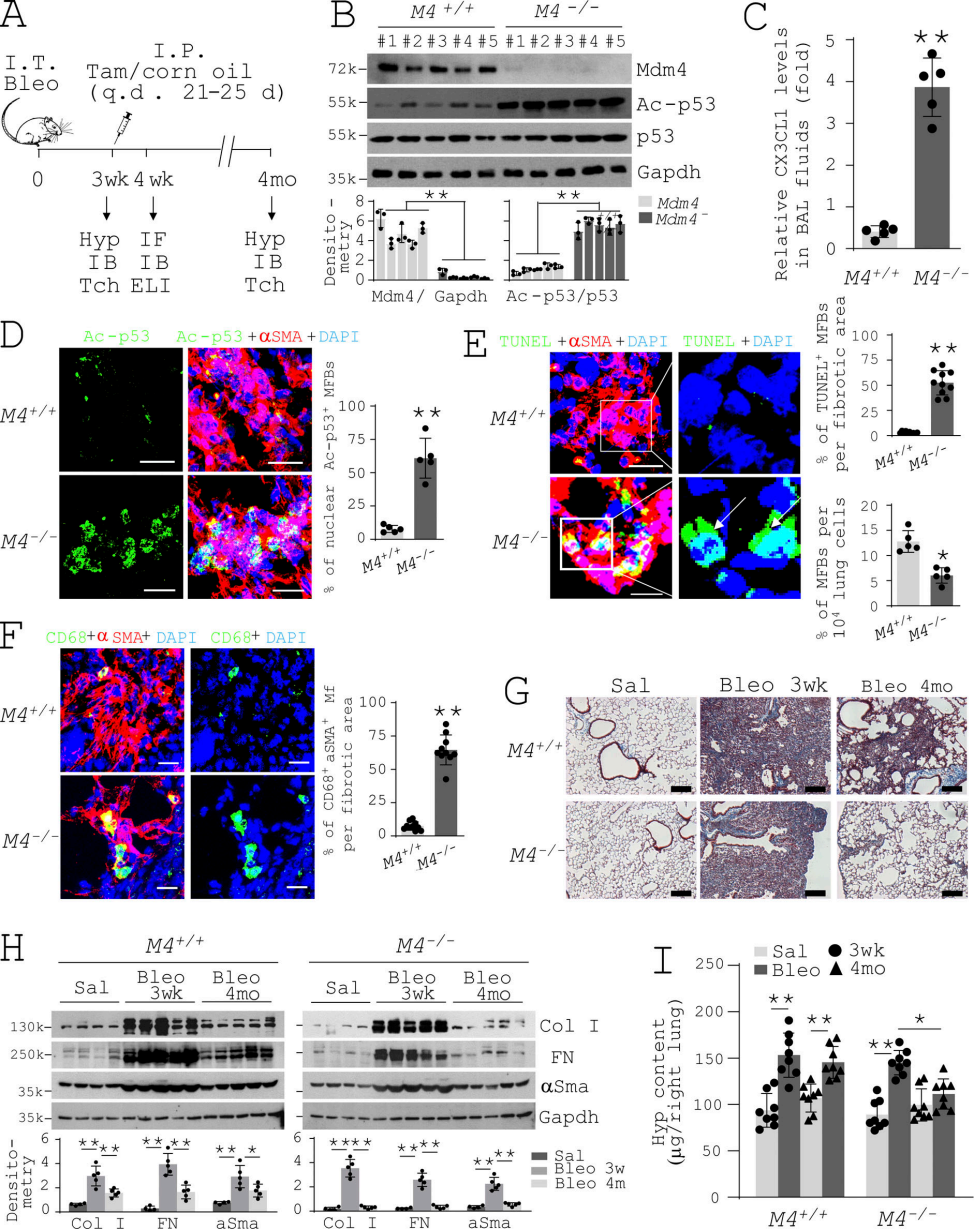
**Genetic deletion of *Mdm4* in mesenchymal cells promotes lung fibrosis resolution in aged mice. (A)** Schematic depiction of the course of animal experiments. ELI, ELISA; Hyp, Hyp content assay; IB, immunoblot; I.P., intraperitoneal; q.d., *quaque die*; Tam, tamoxifen; Tch, trichrome staining. **(B)** Primary lung (myo)fibroblasts were isolated from the fibrotic Col1α2-Cre^ERT2^;Mdm4^fl/fl^ mice treated by tamoxifen or corn oil at week 4 after bleomycin administration. Protein levels of Mdm4 (M4), Ac-p53, and total p53 were determined by immunoblot. GAPDH was used as loading control. **(C)** Levels of CX3CL1 in the BAL fluids of *M4^+/+^* and *M4^−/−^* mice were quantified by ELISA (*n* = 5 per group). **(D)** Ac-p53 expression in αSMA-expressing myofibroblasts in the fibrotic lungs of *M4^+/+^* and *M4^−/−^* mice were evaluated by confocal IF microscopy. Scale bars = 50 µm. **(E)** Apoptosis of lung myofibroblasts (arrows) were evaluated by TUNEL staining and confocal IF microscopy. Scale bars = 50 µm. **(F)** Engulfments of myofibroblasts (αSMA^+^) by macrophage (CD68^+^) were evaluated by confocal IF microscopy. Scale bars = 50 µm. **(G)** Representative images show trichrome staining of lung collagen in paraffin-embedded lung sections harvested from saline- (Sal) or bleomycin- (Bleo) treated *M4^−/−^* and *M4^+/+^* mice at 3 wk and 4 mo. Scale bars = 150 µm. **(H)** Protein levels of collagen I (Col I), fibronectin (FN), and αSMA in mouse lung lysates were determined by immunoblot. GAPDH was used as loading control (*n* = 4–5 mice per group). **(I)** Lung fibrosis resolution in *M4^−/−^* and *M4^+/+^* mice was assessed by decreases in lung Hyp contents comparing 3 wk to 4 mo in the bleomycin-treated group versus the control (saline) group (*n* = 5 mice per group). Quantitative IF analysis was performed in 5–10 randomly selected lung areas from a total of five mice per group. *, P < 0.05; **, P < 0.01 (ANOVA). k, kilodalton.

To determine the role of mechanosensitive MDM4 in lung fibrosis resolution in aged mice, we generated *Mdm4* conditional KO mice (*Col1α2-Cre^ERT2^*;*Mdm4^fl/fl^*), in which tamoxifen-induced expression of Cre recombinase under the control of mouse type I collagen α2 promoter/enhancer–driven specific deletion of *Mdm4* in collagen I–producing cells. Since (myo)fibroblasts synthesize collagen I more than other collagen-positive cells in the fibrotic lungs, gene KO phenotype in the conditional KO mice would be primarily concentrated in lung (myo)fibroblasts relative to other cell types. Conditional KO of *Mdm4* was conducted in 15-mo-old mice at 3 wk following bleomycin administration (Fig. 10 A). Deletion/reduction of Mdm4 expression in lung (myo)fibroblasts isolated from aged mice was confirmed by immunoblot (Fig. 10 B). We found that primary lung (myo)fibroblasts isolated from *Mdm4^−/−^* mice expressed higher levels of acetylated p53 than cells isolated from *Mdm4^+/+^* mice (Fig. 10 B), indicative of p53 activation. Consistent with this result, confocal IF microscopy demonstrated increased acetylated p53-positive signals in the nuclei of lung myofibroblasts in *Mdm4^−/−^* mice as compared with *Mdm4^+/+^* mice (Fig. 10 D). *Mdm4^−/−^* mice expressed a higher level of CX3CL1 in the BAL fluids compared with *Mdm4^+/+^* mice (Fig. 10 C). *Mdm4^−/−^* mice displayed TUNEL-positive nuclei in αSMA-expressing myofibroblasts and reduction in the percentage of myofibroblasts in the lung, suggesting that lung myofibroblasts undergo apoptosis (Fig. 10 E), whereas few apoptotic lung myofibroblasts were observed in *Mdm4^+/+^* mice. Macrophages were found within the αSMA-positive fibrotic lesions in *Mdm4^−/−^* mice, suggestive of macrophage phagocytosis of apoptotic lung myofibroblasts (Fig. 10 F). In contrast, fewer macrophages were found in *Mdm4^+/+^* mice, and the phagocytes were primarily located in the periphery of fibrosis. Histological analysis demonstrated that lung collagen was reduced and the morphology of distal lungs was improved in *Mdm4^−/−^* mice compared with *Mdm4^+/+^* mice (Fig. 10 G). Protein levels of type I collagen, fibronectin, and αSMA in mouse lungs decreased more dramatically at 4 mo compared with 3 wk in *Mdm4^−/−^* mice than *Mdm4^+/+^* mice (Fig. 10 H). Hyp content assays showed that *Mdm4^−/−^* mice had greater reduction (ΔHyp = 38 ± 13 µg) in lung collagen in this period of time than *Mdm4^+/+^* mice (ΔHyp = 11 ± 10 µg; Fig. 10 I). Taken together, these data suggest that genetic ablation of *Mdm4* in lung (myo)fibroblasts activates the p53 pathway and ameliorates persistent lung fibrosis in aged mice.

## Discussion

In this study, we identified MDM4 as a matrix stiffness–regulated mechanosensitive inhibitor of p53. High levels of MDM4 were expressed in the fibrotic lesions of human IPF and experimental lung fibrosis in aged mice. In vitro studies showed that reducing matrix stiffness down-regulates MDM4 expression, resulting in p53 acetylation/activation in primary human lung myofibroblasts isolated from patients with IPF. Gain of p53 function sensitizes lung myofibroblasts to apoptosis by up-regulation of Fas and induces an immunogenic conversion of lung myofibroblasts to release CX3CL1, which recruits macrophages, and to express DD1α, which facilitates phagocytosis of apoptotic myofibroblasts by macrophages. Matrix stiffness–regulated MDM4 expression and p53 activation are preserved in both normal and IPF lung (myo)fibroblasts, suggesting that the cell responses are predominantly governed by mechanical signals from the ECM rather than the origin of the fibroblasts (normal versus fibrotic). In vivo studies provided evidence that destiffening of the ECM by targeting nonenzymatic AGE cross-linking activated the Mdm4–p53-dependent pathway, resulting in accelerated lung fibrosis resolution in aged mice. Genetic ablation of mouse *Mdm4* in lung (myo)fibroblasts reverses persistent lung fibrosis associated with aging.

Previous studies revealed cell type–specific p53 expression in the lung under normal versus fibrotic conditions. p53 expression in epithelial cells increases significantly in usual interstitial pneumonia–like lesions compared with fibrotic nonspecific interstitial pneumonia–like lesions, and fibrotic nonspecific interstitial pneumonia–like lesions compared with normal lung tissues (Jinta et al., 2010). p53 overexpression has been observed at the hyperplastic epithelial foci in honeycomb lesions of IPF (Qunn et al., 2002). AT2 cells in patients with IPF express high levels of p53 in association with DNA strand breaks (Kuwano et al., 1996; Plataki et al., 2005). Consistent with human data, mice exposed to bleomycin lung injury showed marked induction of p53 in AT2 cells (Bhandary et al., 2013; Ghosh et al., 2002). Together, these findings indicate that p53 expression in epithelial cells increases under fibrotic conditions. In contrast, IPF lung fibroblasts express reduced levels of p53 and are more resistant to staurosporine- and S-nitrosoglutathione–induced apoptosis compared with normal fibroblasts (Cisneros et al., 2012). Consistent with this finding, a separate study showed that myofibroblasts at the fibroblastic foci of IPF exhibit negligible p53 expression (Akram et al., 2014). Additionally, alveolar macrophages display p53 translocation from the cytosol to the nucleus, indicative of p53 activation, in response to bleomycin treatment (Davis et al., 2000). In this study, we demonstrated that stiffened matrix up-regulates MDM4 expression in fibrogenic lung myofibroblasts, which mediates p53 repression. We showed that reducing matrix stiffness down-regulates MDM4 expression, which derepresses and activates p53 through induction of protein acetylation at Lys382 residue. The mechanisms by which MDM4 regulates p53 acetylation remain elusive. Previous studies have shown that p300/CREB-binding protein (CBP) histone acetyltransferase promotes p53 acetylation at Lys382 (Gu and Roeder, 1997). Interestingly, both MDM4 and p300/CBP bind p53 in a mutual area at the N terminus (Böttger et al., 1999; Liu et al., 2003; Sabbatini and McCormick, 2002). It is plausible that MDM4 might inhibit p53 acetylation by competing with p300/CBP for binding of p53. MDM4 contains a highly acidic domain in the central region (215–255 aa; Marine et al., 2007). It has been shown that the acidic domain of MDM2 inhibits p300-mediated p53 acetylation (Wang et al., 2008). Additionally, the acidic domain of inhibitor of acetyltransferases complex negatively regulates p300/CBP acetyltransferase activity (Seo et al., 2001). It remains to be determined whether MDM4 antagonizes the acetyltransferase activity of p300/CBP through it acidic domain.

Our studies suggest that stiff matrix up-regulates MDM4 expression by induction of phosphorylation and transactivation of ELK1. Previous studies have shown that mechanical forces promote ELK1 phosphorylation by a RhoA/Rho kinase (ROCK)–dependent signal (Hong et al., 2010; Laboureau et al., 2004). RhoA/ROCK signal activates ERK in response to mechanical stretch (Hong et al., 2010; Khatiwala et al., 2009). Activation of ERK promotes ELK1 phosphorylation (Gille et al., 1995; Jacobs et al., 1999; Yang et al., 1999). In our previous studies, we have demonstrated that stiff matrix activates RhoA/ROCK in human lung fibroblasts (Huang et al., 2012; Zhou et al., 2013). In addition to protein kinase-dependent phosphorylation, the transcriptional activity of ELK1 is also regulated by calcineurin phosphatase–dependent dephosphorylation and/or sumoylation (Sugimoto et al., 1997; Tian and Karin, 1999; Yang et al., 2003). It has been shown that sumoylation in the R domain relocalizes ELK1 to the cytoplasm, resulting in transcriptional suppression (Yang et al., 2002). In this study, we found that matrix stiffness regulates nuclear–cytoplasmic shuttling of ELK1. Future investigations will determine the role of RhoA/ROCK signal and/or calcineurin phosphatase in matrix stiffness–dependent ELK1 transactivation and MDM4 expression.

In this study, we found that reducing matrix stiffness promotes MDM4–p53-dependent Fas expression, which sensitizes lung myofibroblasts to FasL (CH11)–induced apoptosis. In vivo studies provided evidence that destiffening of the fibrotic ECM in aged mice by CA treatment activates the MDM4–p53 pathway and promotes lung myofibroblast apoptosis. However, an in vivo trigger of lung myofibroblast apoptosis remains to be determined. TNFα is involved in elimination of (myo)fibroblasts through Fas-dependent apoptosis (Frankel et al., 2006; Wynes et al., 2011). Previous studies have shown that TNFα accelerates lung fibrosis resolution in mice in a bleomycin injury model (Fujita et al., 2003; Redente et al., 2014). These findings suggest that TNFα might be a potential trigger responsible for matrix destiffening–induced lung myofibroblast apoptosis in vivo. It is known that p53 regulates a number of apoptotic checkpoints both in the extrinsic and the intrinsic pathways (Fridman and Lowe, 2003). Matrix destiffening–induced lung myofibroblast apoptosis may not limit to p53-depedent Fas expression but involves other pathways and/or mechanisms.

Recognition and removal of apoptotic cells are crucial for a healthy repair of injured tissues and reinstatement of tissue homeostasis. Failure to clear apoptotic cells can result in accumulation of autoantigen, giving rise to chronic inflammation and autoimmunity (Muñoz et al., 2010; Nagata, 2010). Macrophages are a major type of phagocytes that play a pivotal role in both progression and resolution of pulmonary fibrosis (Wynn and Vannella, 2016). It has been found that depletion of macrophages in the recovery phase impairs lung fibrosis resolution in mice following bleomycin-induced lung injury (Gibbons et al., 2011), suggesting that macrophages are essential for the reversal of lung fibrosis. TNFα-induced mouse lung fibrosis resolution is associated with reduced numbers of alternatively activated macrophages as well as reprogramming of alternatively activated macrophages into “restorative” macrophages (Fujita et al., 2003; Redente et al., 2014), similar to what has been observed in spontaneous resolution of hepatic fibrosis (Ramachandran et al., 2012). In this study, we showed that MDM4-dependent p53 activation promotes the release of CX3CL1 chemokine by lung myofibroblasts, which functions as a paracrine signal to recruit macrophages. Furthermore, MDM4–p53-dependent DD1α expression facilitates macrophage-mediated phagocytosis of apoptotic lung myofibroblasts. These findings suggest that matrix destiffening activates a MDM4–p53-dependent gene program that promotes immunogenic conversion of lung myofibroblasts. The release of CX3CL1 would serve as a “find-me” signal to recruit macrophages and expression of DD1α as an “eat-me” signal to engulf apoptotic myofibroblasts. An early study has shown that expression of phosphatidylserine (PtdSer) by dying cells involves the clearance of apoptotic cells by phagocytes (Fadok et al., 1992). PtdSer engages in apoptotic cell engulfment by interacting with milk fat globule–epidermal growth factor 8 (MFG-E8; Hanayama et al., 2002). MFG-E8 also promotes macrophage uptake of collagen in mouse lung fibrosis (Atabai et al., 2009). It has been shown that p53 activation promotes PtdSer externalization (Geske et al., 2001; Schuler et al., 2000) and CX3CL1 up-regulates MFG-E8 in apoptotic cells (Leonardi-Essmann et al., 2005; Miksa et al., 2007). These findings suggest that PtdSer and MFG-E8 could potentially contribute to MDM4–p53-dependent clearance of lung myofibroblasts during matrix destiffening–induced lung fibrosis resolution.

Our studies show that MDM4–p53-dependent expression of CX3CL1 by lung myofibroblasts functions as a paracrine signal for recruitment of macrophages. It has been shown that CX3CL1 recruits natural killer (NK) cells, which protects mice against bleomycin-induced lung fibrosis (Jiang et al., 2004). Furthermore, NK cells are involved in liver fibrosis resolution by selectively recognizing and killing hepatic stellate cell–derived myofibroblasts (Melhem et al., 2006; Radaeva et al., 2006). Thus, the role of CX3CL1 in lung fibrosis resolution may not be limited to the recruitment of macrophages but may involve other immune cells, including NK cells.

It has been reported that patients with IPF had higher levels of AGE production in the lung and blood than control subjects (Kyung et al., 2013; Machahua et al., 2016). Diabetes mellitus, which is a group of metabolic diseases characterized by hyperglycemia leading to nonenzymatic glycation of proteins, has been identified as a risk factor of IPF (Ehrlich et al., 2010; Enomoto et al., 2003; García-Sancho Figueroa et al., 2010; Gribbin et al., 2009; Kim et al., 2012b). These findings suggest a role of protein glycation in the pathogenesis of IPF. In this study, we found that nonenzymatic AGE cross-linking is increased in aged lungs and occurs at an accelerated rate in lung fibrosis. Targeting nonenzymatic AGE cross-linking by CA, a potent breaker and inhibitor of AGE cross-linking that exerts its function by reactions with methylglyoxal and dicarbonyl intermediates in the Maillard reaction and by its antioxidant and chelating effects (Aldini et al., 2013; Nagai et al., 2012), destiffens fibrotic lungs and promotes lung fibrosis resolution in aged mice. Importantly, treatment of IPF lung tissue slices by CA increases MMP1-mediated degradation of lung collagen ex vivo, presumably by exposure of hidden protease cryptic sites within highly cross-linked triple helical structures (Hoop et al., 2017; Perumal et al., 2008). These findings suggest that targeting nonenzymatic AGE cross-linking is a novel strategy to treat persistent lung fibrosis associated with aging. We observed that CA treatment decreased lung stiffness in aged mice in the saline-treated control group. Although aged mice subjected to CA treatment did not show abnormal lung morphology at least at 4 mo following CA treatment, the potential adverse effects of CA treatment on lung health should be examined thoroughly and in a longer period of time. Another limitation of the current study is that lung cross-links in human tissues were examined in diseased aged samples versus younger donor controls. It cannot be determined whether the observed differences of lung cross-links are age or disease related or both. However, normal lungs we obtained were largely derived from younger donors. It has been shown that CA inhibits urban particulate matter–induced epithelial-to-mesenchymal transition in a human alveolar epithelial cell line (Lee et al., 2019) and reduces reactive oxygen species production and collagen accumulation in a human hepatic stellate cell line (Koo et al., 2016), suggesting that CA may play a multifaceted role in antifibrotic therapies.

Targeting the mechanical properties of lung microenvironment represents a promising strategy for antifibrotic therapy (Tschumperlin and Lagares, 2020). The current study identifies mechanosensitive MDM4 as a novel molecular target in pulmonary fibrosis and highlights the therapeutic potential of targeting nonenzymatic AGE cross-linking to reverse persistent lung fibrosis associated with aging.

## Materials and methods

### Antibodies and reagents

Anti-MDM4 antibodies were purchased from Bethyl Laboratories. Anti-MDM2 antibody was purchased from R&D Systems. Anti–acetylated p53, anti-p53, anti–phospho ELK1, anti-ELK1, and anti–lamin B antibodies were from Cell Signaling. Anti-αSMA antibody was from American Research Products. Anti-Fas (CH11), anti-DD1α, anti-CD68, and anti–collagen I antibodies were from Thermo Fisher Scientific. Anti-AGE antibody was from Abcam. Anti-fibronectin and anti-GAPDH antibodies were from Santa Cruz Biotechnology. CXCL10 neutralizing antibody was from Thermo Fisher Scientific. CX3CL1 neutralizing antibody was from R&D Systems. The activities of commercial CXCL10 and CX3CL1 neutralizing antibodies were verified by functional blocking of cell chemotaxis. C646 and tamoxifen were from Sigma. CA was from MedChem Express. Validated MDM4 siRNAs were from OriGene.

### Isolation of lung (myo)fibroblasts

Primary human and mouse lung (myo)fibroblasts were isolated and characterized as described in our previous study (Chen et al., 2016). Human lung (myo)fibroblasts were isolated from fresh tissues of patients undergoing lung transplantation for IPF or failed donors. The studies involving human subjects were approved by the Institutional Review Board at the University of Alabama at Birmingham. Lung (myo)fibroblasts were maintained in DMEM supplemented with 10% FBS and used at passages 3–5.

### Preparation of PA hydrogels and lung fibroblast–derived ECM with varying stiffness

PA gels with tunable stiffness were fabricated using a published protocol (Tse and Engler, 2010). Gel surfaces were coated with 0.1 mg/ml rat tail collagen I (BD Biosciences). Normal lung fibroblast–derived ECM (C-ECM) was prepared as described previously (Subbiah et al., 2016). C-ECM was decellularized and were treated by 0% or 2% genipin (Sigma) to create soft and stiffened C-ECM (Qu et al., 2018).

### Quantitative real-time PCR

Total RNA was isolated using TRIzol reagent (Invitrogen). 1 µg total RNA was reversely transcribed into cDNA with a cDNA Synthesis Kit (Thermo Scientific). Quantitative PCR (qPCR) reactions were performed using iQ SYBR Green PCR Supermix (Bio-Rad Laboratories) on a LightCycler 480 (Roche Applied Science). Each sample was run in triplicate. Relative quantification was calculated using the comparative *C*_T_ method (Livak and Schmittgen, 2001). Delta CT values of target gene were normalized to GAPDH.

### Subcellular fractionation, coimmunoprecipitation, immunoblot, and densitometry analysis

Nuclear proteins and cytoplasmic proteins were isolated using NE-PER Nuclear and Cytoplasmic Extraction Kit (Thermo Fisher Scientific) as described in our previous study (Huang et al., 2012). Cell lysates containing 10–40 µg total proteins were loaded onto SDS-PA gels under reducing conditions. After electrophoresis, proteins were electrophoretically transferred from the gels to nitrocellulose at 100 V for 1.5 h at 4°C. Membranes were blocked in casein solution (1% casein and 25 mM Na_2_HPO_4_, pH 7.1) for 1 h at room temperature. Primary antibodies were diluted in TBS–Tween 20 and casein solution (1:1) at a working concentration recommended by manufactures. Membranes were incubated with primary antibodies at room temperature for 1 h. After extensive washing, membranes were incubated with peroxidase-conjugated secondary antibodies (0.1 µg/ml) diluted in TBS–Tween 20 for 1 h at room temperature. Immunodetection was performed by chemiluminescence. Blot images were scanned. Bands were quantified by ImageJ (National Institutes of Health).

### ELISAs

An equal number (2 × 10^6^) of human lung myofibroblasts were cultured on PA gels with various stiffness. Cells were washed three to five times with PBS and incubated with freshly added serum-free DMEM containing penicillin–streptomycin for 48 h at 37°C in 5% CO_2_. Supernatant was collected, centrifuged at 4°C at 3,000 *g* for 3 min followed by 5 min at 1,500 *g*, and filtered through 0.2-µm filters. Protein levels of CX3CL1 and CXCL10 in supernatants were quantified using ELISA kits according to the manufacturer’s protocol (R&D Systems). The binding of ELK1 immobilized oligonucleotide containing EBMs was quantified by TransAM ELK-1 Transcription Factor ELISA Kit (Active Motif). Absorbance was measured at 450 nm with an Epoch microplate reader (BioTek).

### DNA cloning and promoter activity assay

Approximately 1 kb of human *MDM4* promoter fragment (−933 nt to +80 nt) containing the EBMs was amplified by genomic PCR using a pair of primers (forward 5′-GCC​TCC​TAA​GTA​GCT​GGG​ATT​A-3′ and reverse 5′-GGT​GGC​AGT​CAC​AGA​TCC​TA-3′). The fragment was cloned into pGL3-basic luciferase reporter vector (Promega). The recombinant vectors were verified by DNA sequencing. 4 µg *MDM4* promoter constructs was cotransfected with 50 ng Renilla luciferase–expressing control vector (Promega). Luciferase activities were determined using a Dual-Luciferase reporter assay system (Promega). Relative light units were measured by an Orion Microplate Luminometer. The firefly luciferase activity corresponding to *MDM4* promoter was normalized to Renilla luciferase activity of the same sample. Results are expressed as fold changes compared with the mean firefly/Renilla ratio of the cells cultured on soft gels.

### Construction of MDM4-expressing lentiviruses and cell infection

The full-length cDNA of human *MDM4* corresponding to NCBI Reference Sequence NM_002393.5 was amplified by PCR with a pair of primers (5′-ATG​ACA​TCA​TTT​TCC​ACC​TCT​G-3′ and 5′-TGC​TAT​AAA​AAC​CTT​AAC​CAG​C-3′). cDNA was subsequently cloned into a lentiviral expression vector, pLJM1-EGFP (Addgene). Viruses were packaged in 293T cells by cotransfection with lentiviral helper plasmids psPAX2 and pMD2.G (both from Addgene). Virus-containing CM was harvested 72 h after transfection, filtered, and used to infect recipient lung myofibroblasts in the presence of 8 µg/ml polybrene (Sigma). Infected cells were selected with 2 µg/ml puromycin (Sigma).

### siRNA-mediated gene knockdown

4 × 10^5^ cells/well (6-well plates) were cultured with 0.5 ml of OptiMEM media (Life Technologies) containing 5 µl Lipofectamine 2000 (Life Technologies) and 50 nM specific siRNAs or scrambled control siRNA (Dharmacon) for 6 h. OptiMEM media were removed, and cells were cultured in 2 ml DMEM supplemented with 10% FBS for 96 h before harvesting.

### TUNEL assay

TUNEL staining was performed using a TdT-fluor in situ apoptosis detection kit (Trevigen). For TUNEL assay in conjugation with IF staining, TUNEL was performed first followed by incubation with the primary antibodies for 24 h. Fluorochrome-conjugated secondary antibodies were used according to the manufacturer’s recommendation. Nuclei were stained with DAPI.

### Flow cytometry

Single-cell suspensions (10^6^ cells) were pelleted and washed once with wash buffer (PBS with 2% FBS and 0.1% NaN_3_) and then incubated with anti-human DD1α antibody or nonimmune isotype control IgG diluted in blocking buffer at a final concentration of 10 µg/ml for 60 min at 4°C. After washing four times with wash buffer, cells were incubated with FITC-conjugated secondary antibody (indirect only) for 60 min at 4°C. After washing four times with wash buffer, stained cells were fixed in PBS containing 1% paraformaldehyde. Flow cytometry was performed on a LSRII Flow Cytometer (BD Biosciences), and data were processed using FACSDiva software (BD Biosciences).

### Macrophage chemotaxis assay

In vitro macrophage chemotaxis assays were performed in 96-Transwell plates (Corning). Approximately 2 × 10^5^ PMA-differentiated THP-1 macrophages or M-CSF mBMDMs were plated in the upper chambers while the lower chambers were filled with the CM. After 6 h of incubation, the medium was aspirated from upper and lower chambers. Chambers were washed once with PBS followed by incubation with calcein-AM at 37°C for 1 h with gentle tapping every 15 min. After removal of the filter, the plates were read with an Epoch microplate reader (485 nm for excitation and 520 nm for emission). Chemotactic effects were presented as chemotaxis index, a ratio of quantitative fluorescence of cells incubated with the CM to cells incubated with serum-free DMEM.

### In vitro phagocytosis assay

Human lung myofibroblasts were cultured on 20-kPa and 1-kPa PA gels in the presence of 500 ng/ml CH11. Apoptotic cells (obtained by flow cytometry cell sorting) were labeled by pHrodo Red (Thermo Fisher Scientific) and were incubated with THP1 at 1:10 to 1:15 ratio (THP1/myofibroblasts). Two hours after coincubation, wells were washed thoroughly with cold serum-free RPMI five times and examined under a Nikon Eclipse Ti fluorescence microscope using bright field or Texas Red filter set. The phagocytic index was calculated using the formula phagocytic index = number of ingested cells/(number of macrophages/100), as described previously (Jaiswal et al., 2009). At least 500 macrophages were counted per well.

### Stepwise collagen solubilization assay

Stepwise collagen solubilization assay was performed as described previously (Popov et al., 2011). 1 g snap-frozen lung tissue was homogenized in 20 ml neutral salt buffer (0.5 M NaCl and 0.05 M Tris, pH 7.5, with protease inhibitor cocktail) and incubated at 4°C overnight on a rotary shaker. After centrifugation at 24,000 *g* for 30 min, the supernatant was collected for collagen determination (salt-soluble collagen). The resulting pellet was extracted with 20 ml 0.5 M acetic acid (acid-soluble collagen), followed by 2 mg/ml pepsin in 0.5 M acetic acid (pepsin-soluble collagen). The remaining insoluble fraction represents mature, highly cross-linked collagen. Each fraction was hydrolyzed in 6 N HCl, concentrated by evaporation, and used to quantify the collagen content via hydroxyproline content assay. The percentage of collagen in each fraction was calculated as fraction of the total hydroxyproline determined in all fractions.

### HPLC

Enzymatic PYD cross-links and nonenzymatic PE cross-links in lung tissues were quantified by single reverse-phase HPLC-fluorescence detection as described previously (Bank et al., 1997). Lung tissue was hydrolyzed in 6 M hydrochloric acid at 110°C for 20 h. After hydrolysis, samples were dried in a vacuum desiccator, redissolved, and then purified with 0.22-µm centrifuge tube filters. The HPLC column was equilibrated with 0.15% (vol/vol) heptafluorobutyric acid in 24% (vol/vol) methanol. Samples were then injected into a Shimadzu HPLC system with spectrofluorometric detector (Shimadzu Corporation). Elution of cross-links and a pyridoxine internal standard (Quidel) was achieved at 40°C at a flow rate of 1.0 ml/min in two steps. Fluorescence was monitored at 0–22 min, 295/400 nm; 22–45 min, 328/378 nm (gain 100; bandwidth 18 mm). Elution of PYD was achieved at 19.09 min and PE at 28.99 min.

### Mechanical testing

The mechanical properties of PA gels, C-ECM, and lung tissues were measured by an MFP-3D-BIO atomic force microscope (Asylum Research) in contact mode. AFM probes were composed of silicon nitride coating with Au (TR400PB, spring constant ranging 10–50 pN/nm; Oxford Instruments Asylum Research). The AFM system was calibrated according to the manufacturer’s instruction before each indentation measurement, and the cantilever spring constant was confirmed by the thermal fluctuation method. Force–indentation profiles were acquired at an indentation rate of 20 µm/s separated by 4 µm spatially in a 16 × 16 sample grid covering a 20 × 20–μm area. The elasticity (Young’s modulus) at each point on the grid was calculated from fitting force–indentation data using a Hertz-based model, F = 2Eδ^2^tan(α)/π(1 − ν^2^), where indentation force (*F*) was calculated by using Hooke’s law (*F* = κΔ*x*), where κ and Δ*x* denote the AFM probe’s spring constant and the probe’s measured deflection, respectively. The indentation depth (*δ*) is calculated from the difference in the *z*-movement of the AFM piezo and the deflection of the probe. *E* is the elastic modulus of lung tissues being studied, and ν denotes the Poisson ratio (0.4). *α* represents the shape of the probes that were considered to be conical with an approximated half-angle of 35 degrees in this report. A contour map was plotted to visualize spatial patterns of elasticity collected in Force-Map mode using MATLAB R2019a (MathWorks). To facilitate the identification of fibrotic tissues, lung tissue sections were stained with a collagen-binding green fluorescence probe (Col-F collagen-binding reagent; ImmunoChemistry Technologies) at 37°C for 1 h followed by two PBS washes at room temperature.

### Animals and experimental protocol

The animal studies were conducted in accordance with the National Institutes of Health Guidelines for Care and Use of Laboratory Animals. Animal usage and protocols were approved by the Institutional Animal Care and Use Committee of the University of Alabama at Birmingham. To generate mesenchymal cell–specific *Mdm4^−/−^* mice, C57BL/6-*Mdm4^fl/fl^* mice (Grier et al., 2006; Valentin-Vega et al., 2009; a gift from a gift from Dr. G. Lozano at UT MD Anderson Cancer Center, Houston, TX) were crossbred with *Col1α2-Cre^ERT2^* mice (The Jackson Laboratory). 15-mo-old conditional *Mdm4^−/−^* mice and wild-type C57BL/6 mice were used in this study. Bleomycin sulfate was dissolved in sterile saline solution and intratracheally instilled into mice by a Stepper Repetitive Pipette (Tridak) as a single dose in 50 µl saline solution per animal (1 U/kg bodyweight). Control mice received 50 µl saline. For tamoxifen (Sigma) treatment, a dosage of 50 mg/kg body weight/d over 5 d or an equal volume of corn oil (vehicle for tamoxifen) was injected intraperitoneally into conditional *Mdm4^−/−^* mice 21 d after bleomycin administration. For CA treatment, a dosage of 25 mg/kg body weight/d or an equal volume of distilled water (vehicle for CA) was given to wild-type C57BL6 mice daily for 14 d consecutively by oral gavage 21 d after bleomycin administration. Mice were sacrificed at 4 mo. Lung tissues were harvested for further analysis.

### Lung histology, IHC, IF, and confocal microscopy

Masson’s trichrome stain for collagen and H&E stain were performed using a kit from Poly Scientific according to the manufacturer’s recommendations. Images were obtained with a Nikon Eclipse TE 300 microscope equipped with Spot Insight charge-coupled device camera and MetaMorph software version 6.2 r4 (Universal Imaging).

IHC was performed as described previously (Zhou et al., 2009). Briefly, 5-µm lung tissue sections were immersed in 10 mmol/liter sodium citrate buffer (pH 6.0) and heated at 100°C for 10 min to unmask antigen. Sections were blocked with 5% normal goat serum and stained with anti-MDM4 antibody at 2.5 µg/ml. Endogenous peroxidase activity was quenched with 3% H_2_O_2_. Staining was developed with biotinylated secondary antibodies, streptavidin–peroxidase, and 3-amino-9-ethylcarbazole chromogen (Vector Laboratories). Semiquantitative analysis of IHC images was performed using ImageJ.

For IF, 30-µm cryostat sections were rehydrated in PBS for 10 min. Tissue sections were blocked with 5% normal goat serum and stained with primary antibodies diluted in PBS containing 1% goat serum, 0.3% Triton X-100, and 0.01% sodium azide according to manufacturer’s instructions. Fluorochrome-conjugated secondary antibodies (SouthernBiotech) were used according to the manufacturer’s recommendation. Nuclei were stained with DAPI (Thermo Fisher Scientific). Fluorescent signals were detected using a confocal laser-scanning microscope Zeiss LSM710 confocal microscope equipped with a digital color camera. All fluorescent images were generated using sequential laser scanning with only the corresponding single-wavelength laser line, activated using acousto-optical tunable filters to avoid cross-detection of either one of the fluorescence channels.

### Hyp content assay

Mouse right lungs were homogenized in 2.0 ml distilled water and incubated with 125 µl of 50% trichloroacetic acid on ice for 20 min. Samples were centrifuged and the pellets were mixed with 1 ml 12 N hydrochloric acid and baked at 110°C for 14–18 h. Dry samples were dissolved in 2 ml deionized water. 200-µl samples (or standards) were added to 500 µl 1.4% chloramine T in 0.5 M sodium acetate/10% isopropanol (Thermo Fisher Scientific) and incubated for 20 min at room temperature. 500 µl Ehrlich's solution (1.0 M p-dimethylaminobenzaldehyde in 70% isopropanol/30% perchloric acid; Thermo Fisher Scientific) was added, mixed, and incubated at 65°C for 15 min. Optical density of each sample and standard was measured at 550 nm, and the concentration of lung Hyp was calculated from a Hyp standard curve.

### Statistical analysis

Experimental results were obtained from biological replicates averaged to generate a single summary value, recorded as mean ± SD under each condition. Statistical differences among treatment conditions were determined using one-way ANOVA. The analysis was performed with GraphPad Prism 8 software. Values of P < 0.05 or P < 0.01 were considered significant.

### Online supplemental material

Fig. S1 shows Mdm4 expression during the course of bleomycin-induced lung fibrosis in aged mice and comparision of Mdm4 expression between uncultured lung fibroblasts and alveolar epithelial cells isolated from aged mice. Fig. S2 shows that MDM4 is a matrix stiffness–regulated mechanosensitive inhibitor of p53. Fig. S3 shows the effects of matrix stiffness on transcription of chemokines in primary lung myofibroblasts isolated from patients with IPF. Fig. S4 shows that nonenzymatic AGE cross-linking is increased in human IPF and experimental lung fibrosis in aged mice. Fig. S5 shows the effects of CA on MDM4 and MDM2 expression, lung myofibroblast apoptosis, and sensitization of lung myofibroblasts to FasL-induced apoptosis.
